# TCF1^+^ hepatitis C virus-specific CD8^+^ T cells are maintained after cessation of chronic antigen stimulation

**DOI:** 10.1038/ncomms15050

**Published:** 2017-05-03

**Authors:** Dominik Wieland, Janine Kemming, Anita Schuch, Florian Emmerich, Percy Knolle, Christoph Neumann-Haefelin, Werner Held, Dietmar Zehn, Maike Hofmann, Robert Thimme

**Affiliations:** 1 Department of Medicine II, University Hospital Freiburg — Faculty of Medicine, University of Freiburg, Hugstetter Straße 55, Freiburg 79106, Germany; 2 Spemann Graduate School of Biology and Medicine, University of Freiburg, Freiburg 79104, Germany; 3 Faculty of Biology, University of Freiburg, Freiburg 79104, Germany; 4 Institute for Cell and Gene Therapy, University Hospital Freiburg, Hugstetter Straße 55, 79106 Freiburg, Germany; 5 Institute of Molecular Immunology and Experimental Oncology, Technische Universität München, Klinikum rechts der Isar, Ismaningerstr. 22, München 81675, Germany; 6 Ludwig Center for Cancer Research, Department of Fundamental Oncology, University of Lausanne, 155, Ch. Des Boveresses, Epalinges 1066, Switzerland; 7 Division of Animal Physiology and Immunology, School of Life Sciences Weihenstephan, Technical University Munich, Freising, Weihenstephaner Berg 3, Freising 85354, Germany

## Abstract

Differentiation and fate of virus-specific CD8^+^ T cells after cessation of chronic antigen stimulation is unclear. Here we show that a TCF1^+^CD127^+^PD1^+^ hepatitis C virus (HCV)-specific CD8^+^ T-cell subset exists in chronically infected patients with phenotypic features of T-cell exhaustion and memory, both before and after treatment with direct acting antiviral (DAA) agents. This subset is maintained during, and for a long duration after, HCV elimination. After antigen re-challenge the less differentiated TCF1^+^CD127^+^PD1^+^ population expands, which is accompanied by emergence of terminally exhausted TCF1-CD127-PD1^hi^ HCV-specific CD8^+^ T cells. These results suggest the TCF1^+^CD127^+^PD1^+^ HCV-specific CD8^+^ T-cell subset has memory-like characteristics, including antigen-independent survival and recall proliferation. We thus provide evidence for the establishment of memory-like virus-specific CD8^+^ T cells in a clinically relevant setting of chronic viral infection and we uncover their fate after cessation of chronic antigen stimulation, implicating a potential strategy for antiviral immunotherapy.

## Abstract (web summary)

Virus-specific CD8^+^ T cells lose effector function over the course of chronic infection, a process called ‘exhaustion', but the fate of these cells after treatment-induced antigen elimination is unknown. Here the authors show that exhausted cells persist in patients even after direct-acting antiviral therapy removes antigen exposure, and that these cells are responsive on re-exposure to antigen.

Human chronic viral infections with hepatitis C virus (HCV), hepatitis B virus (HBV) and human immunodeficiency virus (HIV) are a major global health problem. A rheostat that determines control versus active persistence of these viral infections is the virus-specific CD8^+^ T-cell response^1^,^2^. Virus-specific CD8^+^ T cells are polyfunctional in controlled infection, whereas virus-specific CD8^+^ T-cell function is compromised in actively persisting infection. One important mechanism underlying impaired virus-specific CD8^+^ T-cell responses in human chronic viral infection is the progressive loss of effector functions, a phenomenon called T-cell exhaustion^3^,^4^,^5^. Thus, immunotherapeutic strategies that interfere with virus-specific CD8^+^ T-cell exhaustion and consequently boost polyfunctional CD8^+^ T-cell responses are considered to be promising approaches to combat or prevent chronic viral infections in humans.

Major advances in the understanding of CD8^+^ T-cell exhaustion during chronic viral infection in general have been made using the lymphocytic choriomeningitis virus (LCMV) mouse model. In particular, exhausted CD8^+^ T cells can be defined by a reduced cytokine production, an impaired proliferative capacity, the expression of multiple co-inhibitory molecules, the up-regulation of ectonucleotidase CD39 and an altered global transcriptional program and epigenetic profile^6^,^7^,^8^,^9^. Several of these characteristics have also been reported for exhausted virus-specific CD8^+^ T cells in human chronic infections including functional impairment, co-expression of inhibitory receptors and the increased expression of CD39 and the transcription factor Eomes^10^,^11^,^12^,^13^,^14^. Importantly, in chronic LCMV infection, exhausted virus epitope-specific CD8^+^ T-cell populations are not homogeneous. Two subsets of exhausted LCMV epitope-specific CD8^+^ T cells are defined by differential levels of the inhibitory receptor PD1 and the two transcription factors Tbet and Eomes^15^. Tbet^hi^Eomes^dim^PD1^int^ LCMV-specific CD8^+^ T cells are progenitor cells that can give rise to terminally exhausted Tbet^dim^Eomes^hi^PD1^hi^ cells. Both progenitor and terminal subsets of exhausted LCMV epitope-specific CD8^+^ T cells are required to sustain viral control during viral persistence^15^. With respect to chronic infections in humans, however, our knowledge about subsets, differentiation and maintenance of virus-specific CD8^+^ T cells is limited and efficient immunotherapeutic approaches are required.

Although, the mechanisms responsible for CD8^+^ T-cell exhaustion are not completely understood, an important feature seems to be prolonged and continuous exposure to antigen and, consequently, progressive terminal differentiation^16^,^17^,^18^. Additional factors, including lack of CD4^+^ T-cell help, immunosuppressive cytokines and instructive signals directly from inhibitory receptors also contribute to T-cell exhaustion^6^,^19^,^20^. Remarkably, blockade of the PD1/PDL1 inhibitory pathway leads to functional restoration of exhausted virus-specific CD8^+^ T cells^21^,^22^,^23^. Therefore, despite ongoing antigen recognition and consequently progressive terminal differentiation, functional T-cell exhaustion, in principle, is reversible. Importantly, only a distinct sub-population of less differentiated PD1^+^ virus-specific CD8^+^ T cells is rescued by blockade of the PD1/PDL1 pathway in chronic LCMV infection, whereas terminally exhausted subsets do not respond well^24^. PD1^+^ LCMV-specific CD8^+^ T cells that provide the proliferative burst after PD1/PDL1 pathway blockade are characterized by CXCR5 and TCF1 expression and by a unique gene signature^25^,^26^,^27^,^28^. Interestingly, this LCMV-specific CD8^+^ T-cell population possesses self-renewal capacity, gives rise to terminally exhausted effector subsets and therefore sustains the virus-specific CD8^+^ T-cell pool during antigen persistence. Furthermore, the LCMV-specific TCF1^+^CD8^+^ T-cell subset readily expands after transfer into naive mice and upon re-challenge with LCMV, suggesting memory-like characteristics^27^.

The fate of exhausted virus-specific CD8^+^ T cells after cessation of chronic antigen stimulation in a previously persistently infected organism has not been defined - neither in mice nor in humans. In the LCMV mouse model, drugs that efficiently eliminate the virus are not available. The same holds true for human persistent infections with the exception of IFNα-based therapies for chronic viral hepatitis. IFNα has a known immunomodulatory effect during therapy, rendering studies of immune function difficult to interpret^29^,^30^. The fate of exhausted virus-specific CD8^+^ T cells after removal of persistent antigen, however, is of central clinical relevance since it has implications for protection from re-infection after antigen elimination. By approval of direct acting antiviral (DAA) agents for HCV therapy that are highly efficient at viral elimination this important question can now be addressed for the first time. Here we take advantage of a well-defined cohort of chronically HCV-infected patients who have been treated successfully with DAAs. We comprehensively study virus-specific CD8^+^ T-cell responses during and after chronic antigen exposure at a single-cell level. Importantly, we identify a CD127^+^PD1^+^ population of exhausted HCV epitope-specific CD8^+^ T cells during antigen persistence that is maintained long-term after cessation of chronic antigen stimulation. This CD127^+^PD1^+^ subset is further characterized by the expression of TCF1 and BCL2 and thus shows phenotypic features of both T-cell memory and exhaustion. When re-stimulated *in vitro* the TCF1^+^CD127^+^PD1^+^ subset contains the proliferative capacity of the HCV-specific CD8^+^ T-cell population both during and after antigen persistence. Moreover, this subset expands in response to viral relapse *in vivo*. In sum, TCF1 defines a CD127^+^PD1^+^ subset of HCV-specific CD8^+^ T cells that exhibits memory-like characteristics and therefore appear to be a promising target to boost HCV-specific CD8^+^ T cell responses for immunotherapeutic interventions and protection from re-infection.

## Results

### T-cell heterogeneity during persistent antigen recognition

**Figure 1:**
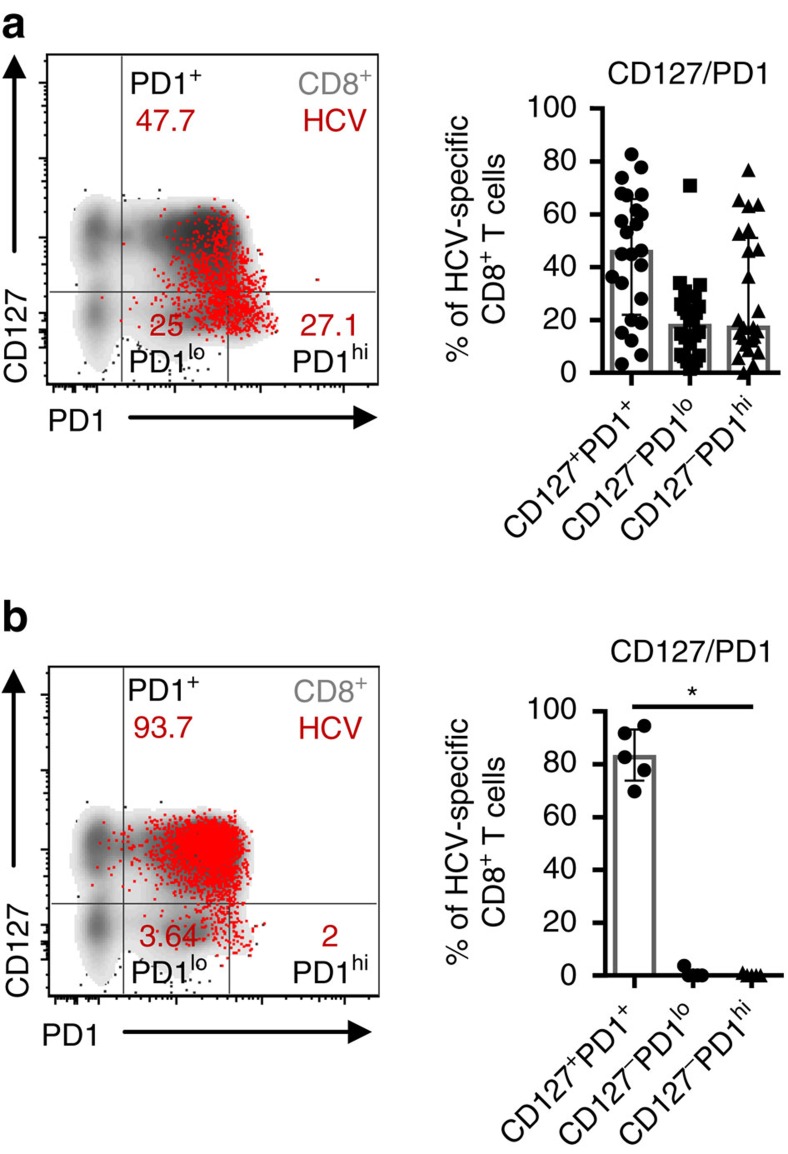
Persistent antigen recognition drives heterogeneity of HCV-specific CD8^+^ T cells. (**a**) CD127/PD1 co-expression analysis of 24 HCV epitope-specific CD8^+^ T-cell populations derived from 19 chronically HCV-infected patients targeting autologous viral epitopes. Subset definition, specifically CD127^+^PD1^+^, CD127-PD1^lo^ and CD127-PD1^hi^ cells, is depicted in the representative flow cytometry dot plot on the left (red: HCV epitope-specific CD8^+^ T cells; grey: corresponding bulk CD8^+^ T cells of the appropriate patient). (**b**) Analysis of CD127/PD1 co-expression of five HCV epitope-specific CD8^+^ T-cell populations from five chronically HCV-infected patients that do not recognize viral antigen due to viral escape mutations. Representative dot plot on the left shows HCV epitope-specific (red) and bulk (grey) CD8^+^ T cells. Bar charts show the median value with interquartile range. Friedman and Dunn's multiple comparison tests were applied for paired statistical analyses. (*P<0.05)

In chronic HCV infection, the expression levels of the inhibitory receptor PD1 and the IL7R α-chain, CD127, have been proposed to discriminate between virus-specific CD8^+^ T cells that are exhausted due to persistent recognition of autologous viral epitopes and virus-specific CD8^+^ T cells that do not recognize autologous viral epitopes due to the emergence of viral escape mutations^10^,^31^. Indeed, HCV-specific CD8^+^ T-cell populations that predominantly express CD127 indicate the presence of viral sequence variations in the corresponding viral epitope. In contrast, in the absence of viral sequence variations and thus ongoing antigen triggering, HCV-specific CD8^+^ T cells exhibit high expression of PD1, co-expression of additional inhibitory receptors and low proliferative capacity, suggesting exhaustion of this T-cell population. However, whether an exhausted HCV-specific CD8^+^ T-cell population targeting a single epitope is a homogeneous population has not been analysed previously. To address this question we performed co-expression analyses of PD1 and CD127 on single HCV epitope-specific CD8^+^ T cells and applied the peptide/MHCI tetramer-associated magnetic bead enrichment technique to allow high sensitivity HCV epitope-specific CD8^+^ T-cell characterization (Supplementary Fig. 1). Peptide/MHCI tetramers specific for three well-described HLA-A*02-restricted epitopes (NS3_1073_; NS3_1406_; NS5_2594_) were utilized and naive HCV epitope-specific CD8^+^ T cells were excluded from the analyses (Supplementary Fig. 2). CD127/PD1 co-expression of 24 HCV epitope-specific CD8^+^ T-cell populations derived from 19 chronically HCV-infected patients that target autologous viral epitopes (Table 1) is depicted in Fig. 1a with a representative dot plot shown on the left. Interestingly, most HCV epitope-specific CD8^+^ T-cell populations did not display a homogenous but rather a heterogeneous phenotype that included CD127^+^PD1^+^ (Mdn: 45.7%; IQR: 22.0-65.7%), CD127-PD1^lo^ (Mdn: 17.7; IQR: 6.6-25.7%) and CD127-PD1^hi^ (Mdn: 17.1; IQR: 11.0-51.1%) subsets, rendering a subset of the HCV epitope-specific CD8^+^ T cells responsive to the T-cell maintenance factor IL-7.

To determine whether this heterogeneous phenotype of HCV epitope-specific CD8^+^ T cells is linked to persistent antigen recognition, we next analysed CD127 and PD1 co-expression on 5 HCV epitope-specific CD8^+^ T-cell populations derived from 5 chronically HCV-infected patients that did not recognize the autologous viral epitope due to the presence of viral escape mutations (Table 1). As shown in Fig. 1b, HCV epitope-specific CD8^+^ T cells that target variant epitopes and are thus not triggered by ongoing antigen recognition do not show strong heterogeneity but rather consist of a predominantly CD127^+^PD1^+^ population (Mdn: 82.7%; IQR: 73.8-93.1%). These results clearly demonstrate that persistent antigen recognition induces heterogeneity of a single HCV epitope-specific CD8^+^ T-cell population and more specifically the occurrence of a CD127-PD1^hi^ HCV epitope-specific CD8^+^ T-cell subset.

### TCF1 defines a memory-like HCV-specific CD8^+^ T-cell subset

**Figure 2:**
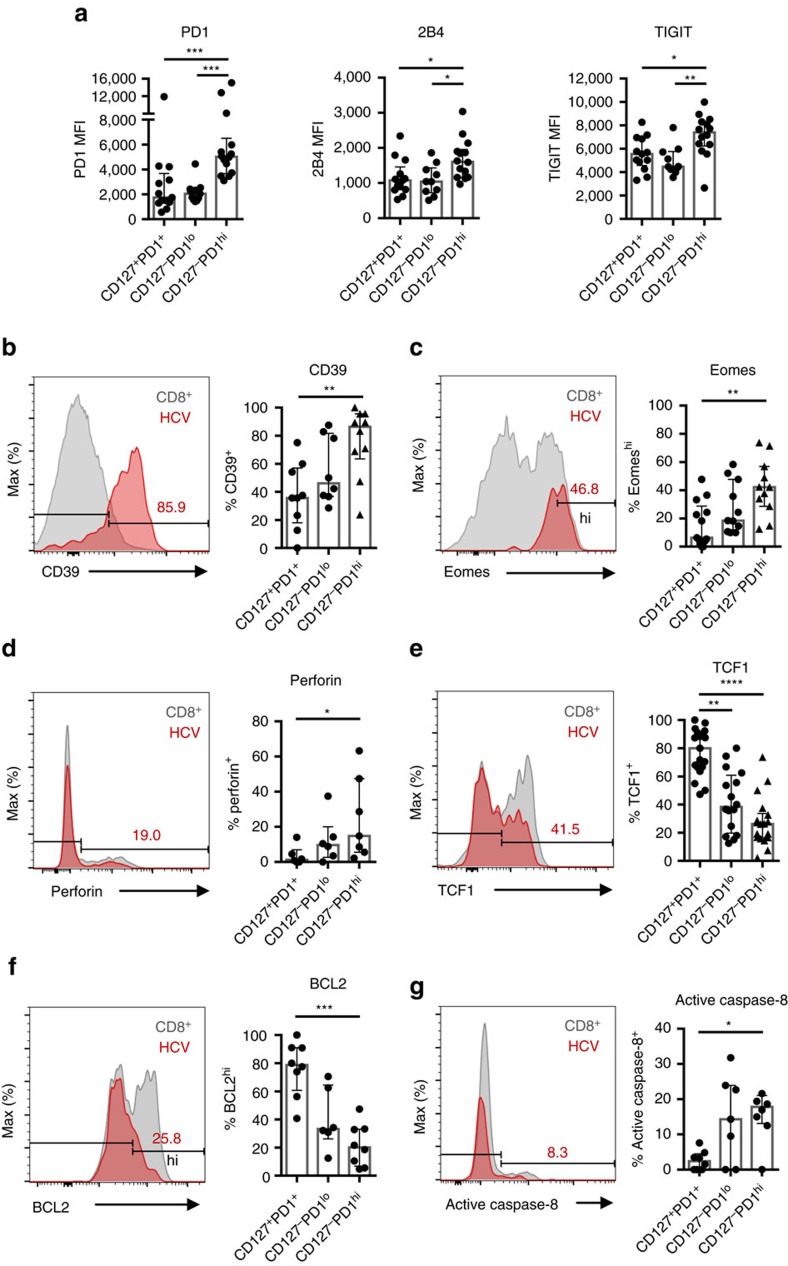
TCF1 defines CD127^+^PD1^+^ HCV-specific CD8^+^ T cells that are less differentiated. HCV epitope-specific CD8^+^ T cells were analysed for expression levels (MFI=median fluorescence intensity) of the inhibitory receptors PD1, 2B4, TIGIT (**a**) and the relative expression of CD39 (**b**), Eomes (**c**), Perforin (**d**), TCF1 (**e**), BCL2 (**f**) and active caspase-8 (**g**) with respect to the CD127/PD1 subsets (n=7–19). Representative flow cytometric histogram plots including gating of the individual markers are depicted (red: HCV epitope-specific CD8^+^ T cells; grey: corresponding bulk CD8^+^ T cells). Bar charts show the median value with interquartile range. Statistical significance was assessed by Kruskal-Wallis with Dunn's multiple comparison test. (*P<0.05; **P<0.01; ***P<0.001; ****P<0.0001.)

Next we set out to define the molecular signatures of the different CD127/PD1 subsets of HCV epitope-specific CD8^+^ T cells. CD127-PD1^hi^ HCV epitope-specific CD8^+^ T cells exhibited increased levels of the inhibitory receptors 2B4 and TIGIT (Fig. 2a), expressed the ectonucleotidase CD39 (Fig. 2b) and displayed high levels of the transcription factor Eomes (Fig. 2c). Thus, this subset displayed typical features of terminal T-cell exhaustion. In contrast, CD127^+^PD1^+^ HCV epitope-specific CD8^+^ T cells expressed low levels of inhibitory receptors, CD39 and Eomes (Fig. 2a–c). Of note, all HCV-specific CD8^+^ T cells are T-bet^dim^ (Supplementary Fig. 3). To further address the differentiation stage of the particular CD127/PD1 subsets, we stained for the effector cell molecule perforin and the transcription factor TCF1 that is required for the differentiation and persistence of memory CD8^+^ T cells^32^,^33^,^34^. While 14.9% (IQR: 5.6–47.4%) of CD127-PD1^hi^ HCV epitope-specific CD8^+^ T cells expressed perforin, CD127^+^PD1^+^ subsets almost completely lacked perforin expression (Fig. 2d). On the other hand, CD127^+^PD1^+^ HCV epitope-specific CD8^+^ T cells harboured the highest proportion of TCF1 expressing cells (Mdn: 80.0%; IQR: 66.5–91.0%) compared to CD127-PD1^lo^ (Mdn: 38.3%; IQR: 19.7–60.8%) and CD127-PD1^hi^ (Mdn: 26.2%; IQR: 16.3–33.7%) subsets (Fig. 2e). Of note, we did not observe expression of CXCR5 on TCF1^+^ HCV epitope-specific CD8^+^ T cells (Supplementary Fig. 4). Altogether, these data suggest that CD127^+^PD1^+^ HCV epitope-specific CD8^+^ T cells represent less differentiated memory-like cells while CD127-PD1^hi^ cells define terminally exhausted effector subsets of HCV epitope-specific CD8^+^ T cells. As shown in Fig. 2f, this hypothesis was further supported by the finding, that similar to memory CD8^+^ T cells CD127^+^PD1^+^ HCV epitope-specific CD8^+^ T cells expressed the anti-apoptotic molecule BCL2 to a great extent (Mdn: 78.8%; IQR: 60.7–91.1%) whereas CD127-PD1^hi^ subsets showed minor expression of BCL2 (Mdn: 20.2%; IQR: 6.8-33.2%). The low expression of BCL2 in this terminal differentiated sub-population was accompanied by high caspase-8 activity (Fig. 2g) suggesting depletion of the CD127-PD1^hi^ sub-population by apoptosis. Interestingly, CD127-PD1^hi^ HCV epitope-specific CD8^+^ T cells are positive for CD122 (Mdn: 84.6%; IQR: 69.1-90.3%; Supplementary Fig. 5) rendering these cells susceptible for IL-15-mediated bystander proliferation as has been reported for effector and effector-memory CD8^+^ T cells.

### Memory-like T cells are maintained antigen-independently

**Figure 3:**
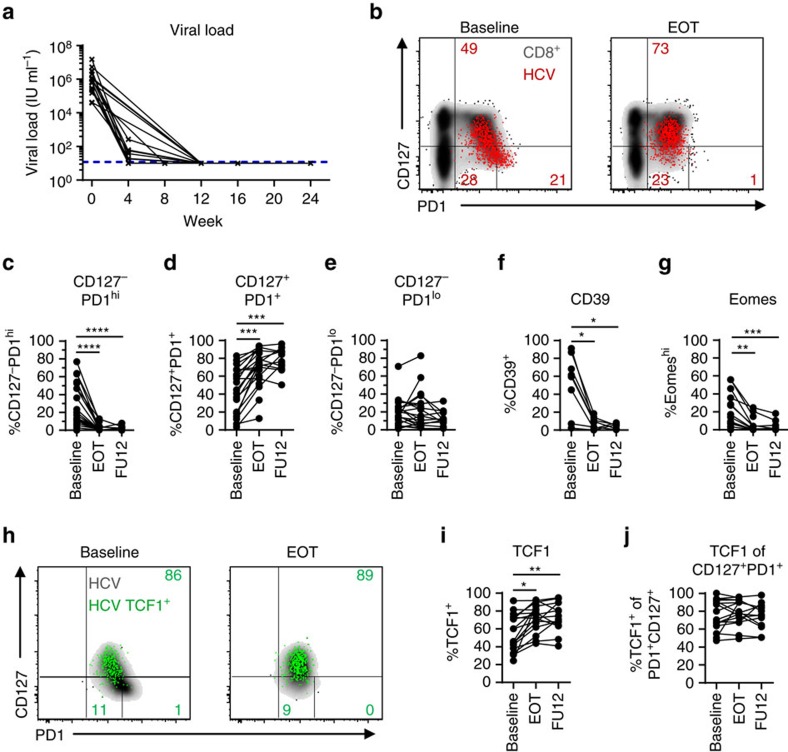
TCF1^+^CD127^+^PD1^+^ HCV-specific CD8^+^ T cells are maintained after cessation of antigen stimulation. CD127/PD1 subset distribution of HCV epitope-specific CD8^+^ T cells after cessation of antigen stimulation by direct acting antiviral (DAA) therapy (n=9–16). (**a**) Viral loads (HCV RNA) in the sera of DAA-treated HCV patients (n=20) were determined at the indicated time-points. Lower detection limit is indicated by dashed line. (**b**) Representative dot plots showing CD127/PD1 subset distribution of HCV epitope-specific CD8^+^ T cells (red) at baseline (left) and at the end of DAA therapy (EOT; right) (grey: corresponding bulk CD8^+^ T cells). (**c**–**e**) Statistical graphs depicting frequencies of the indicated CD127/PD1 subset among HCV epitope-specific CD8^+^ T cells during DAA-mediated cessation of antigen stimulation. (**f**,**g**) Frequencies of CD39^+^ and Eomes^hi^ HCV epitope-specific CD8^+^ T cells during DAA-mediated cessation of antigen stimulation. (**h**) Representative flow cytometric dot plots depict CD127/PD1 co-expression of TCF1^+^ HCV epitope-specific CD8^+^ T cells (green; grey: corresponding bulk HCV epitope-specific CD8^+^ T cells) at baseline (left) and at the end of DAA therapy (EOT, right). Frequencies of TCF1-expressing HCV epitope-specific CD8^+^ T cells (**i**) and TCF1-expressing CD127^+^PD1^+^ HCV epitope-specific CD8^+^ T cells (**j**) during DAA-mediated HCV elimination. Statistical significance was assessed by Friedman and Dunn's multiple comparison tests. (*P<0.05; **P<0.01; ***P<0.001; ****P<0.0001.)

Antigen-independent survival is a hallmark of memory CD8^+^ T cells in resolved infections. In the LCMV mouse model of chronic infection, antigen-independent maintenance and consequently memory potential of exhausted virus-specific CD8^+^ T cells has also been demonstrated after transfer in naive mice^18^,^35^. However, in humans, very little is known about memory potential including survival characteristics of exhausted virus-specific CD8^+^ T-cell subsets. To assess whether CD127/PD1 subsets reveal differences in survival depending on the presence of antigen we longitudinally analysed HCV epitope-specific CD8^+^ T cells of 22 chronically HCV-infected patients after initiation of DAA therapy (Table 1 and Supplementary Fig. 6). As shown in Fig. 3a and Table 1, DAA therapy led to a rapid decline of viremia early after therapy initiation and to a sustained virological response (SVR), defined as undetectable HCV RNA 12 weeks post treatment in all but one treated patients. The single patient with viral relapse was excluded from the SVR cohort (n=21) and analysed separately. Inhibition of viral replication and most likely removal of viral antigen led to significant dynamic changes in the CD127/PD1 subset distribution within single HCV epitope-specific CD8^+^ T-cell populations (Fig. 3b). As shown in Fig. 3c–e and Supplementary Fig. 7b, we found a dramatic decrease in the frequency of CD127-PD1^hi^ HCV epitope-specific CD8^+^ T cells (Fig. 3c) with a concomitant increase in the frequency of the CD127^+^PD1^+^ subset (Fig. 3d and Supplementary Fig. 8) while the CD127-PD1^lo^ T-cell population remained rather unchanged (Fig. 3e). Of note, we did not observe changes in the overall frequency of HCV epitope-specific CD8^+^ T cells (Supplementary Fig. 7a). In agreement with our finding that the CD127-PD1^hi^ subset represents terminally exhausted T cells, we also observed a significant decrease in the frequencies of HCV epitope-specific CD8^+^ T cells that highly express CD39 (Fig. 3f) and Eomes (Fig. 3g) after initiation of DAA therapy and consequent cessation of chronic antigen stimulation. In addition, initiation of DAA therapy led to a significant relative increase of TCF1^+^ HCV epitope-specific CD8^+^ T cells (Fig. 3h,i) that can be ascribed to the increased frequency of CD127^+^PD1^+^ HCV epitope-specific CD8^+^ T cells since TCF1 expression remained stable within the CD127^+^PD1^+^ subset (Fig. 3h,j and Supplementary Fig. 9). To confirm that the dynamic changes of HCV epitope-specific CD8^+^ T cells induced by DAA therapy are indeed due to the cessation of chronic antigen stimulation we additionally analysed HLA-A*0201-restricted Flu M1_58_ - and CMV pp65_495_-specific CD8^+^ T cells derived from DAA-treated HCV-infected patients (Supplementary Table 1). Importantly, these virus-specific CD8^+^ T cells remained phenotypically stable (Supplementary Fig. 10a–e) clearly indicating that the observed changes in HCV epitope-specific CD8^+^ T cells are linked to cessation of chronic antigen stimulation rather than abrogation of the inflammatory milieu. To further confirm antigen-dependency and to exclude DAA-specific effects, we analysed HCV epitope-specific CD8^+^ T-cell responses of patients successfully treated with IFNα-based therapy. Due to the immunomodulatory effects of IFNα-based therapy, we comapred patient samples acquired before therapy and at a late time point following IFNα administration. The observed changes in CD127/PD1, CD39, Eomes and TCF1 expression of HCV epitope-specific CD8^+^ T cells based on IFNα therapy (Supplementary Table 2 and Supplementary Fig. 10f–k) were similar to the ones observed with IFNα-free DAA therapy. These results confirm the impact of persistent antigen on virus-specific CD8^+^ T cells and the presence of memory-like HCV epitope-specific CD8^+^ T cells that are maintained after antigen elimination.

### Memory-like and conventional memory T cells differ

**Figure 4:**
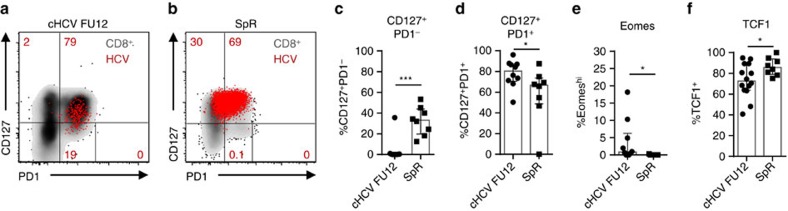
Memory-like and conventional memory HCV-specific CD8^+^ T cells differ. HCV epitope-specific CD8^+^ T cells from patients (*n*=9–10) 12 weeks after the end of DAA therapy (cHCV FU12) were compared to HCV epitope-specific CD8^+^ T cells from patients who spontaneously resolved HCV infection (SpR; *n*=6). Representative flow cytometric dot plots depict CD127/PD1 co-expression of HCV epitope-specific CD8^+^ T cells derived from cHCV FU12 (**a**) and SpR (**b**) (red: HCV epitope-specific CD8^+^ T cells, grey: corresponding bulk CD8^+^ T cell). Frequencies of the indicated CD127/PD1 subset (**c**,**d**), Eomes^hi^ (**e**) and TCF1^+^ cells (**f**) among HCV epitope-specific CD8^+^ T cells present in cHCV FU12 and SpR. Bar charts show the median value with interquartile range. Statistical significance was assessed by Mann—Whitney test. (*P<0.05; ***P<0.001.)

Next, we asked whether the HCV epitope-specific CD8^+^ T-cell populations that are maintained after spontaneous or DAA-mediated viral clearance show a similar phenotype (Table 1). As shown in Fig. 4a-d, the CD127/PD1 subset distribution of HCV epitope-specific CD8^+^ T cells differed between the two cohorts. Specifically, HCV epitope-specific CD8^+^ T cells derived from donors with spontaneous HCV elimination contained a CD127^+^PD1- subset (Mdn: 33.1%; IQR: 19.8-43.9%) that was almost completely absent in HCV epitope-specific CD8^+^ T cells from DAA-treated donors (Fig. 4c). This difference in CD127/PD1 subset distribution was accompanied on a transcriptional level by a lower expression of Eomes (Fig. 4e) and a higher expression of TCF1 (Fig. 4f). Taken together, these results clearly show phenotypical differences between memory-like HCV epitope-specific CD8^+^ T cells that are maintained after DAA-mediated viral clearance and conventional memory HCV epitope-specific CD8^+^ T cells that persist after spontaneous viral clearance. Hence, HCV-specific CD8^+^ T-cell differentiation varies in chronic and acute resolved infection, although persisting memory and short-lived effector subsets are established in both differentiation programs.

### Impaired T-cell effector function after antigen elimination

**Figure 5:**
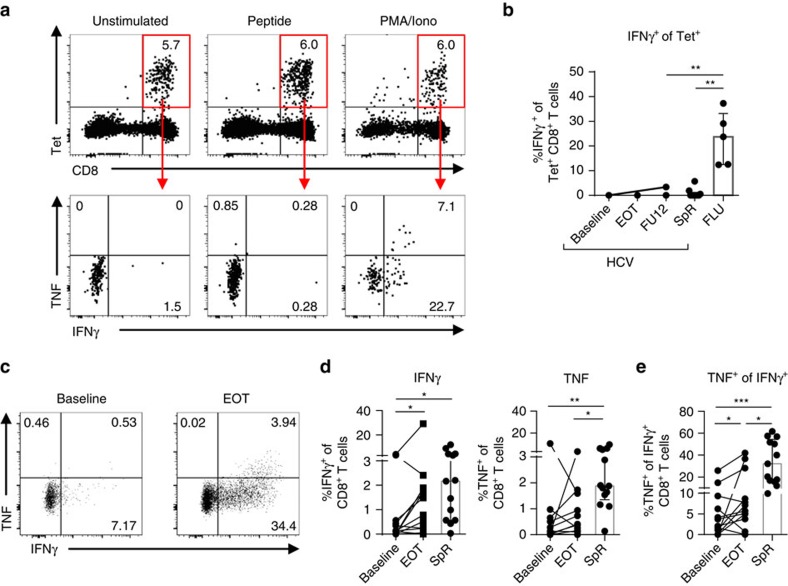
Low cytokine production by memory-like HCV-specific CD8^+^ T cells. Virus-specific CD8^+^ T cells were stimulated with epitope-specific peptides (10 μg ml^−1^) for 5 h at 37 °C and analysed for cytokine production (**a**,**b**). (**a**) Flow cytometric dot plots demonstrate stability of peptide/MHCI tetramer staining (upper row) and IFNγ and TNF production (lower row) of HCV epitope-specific CD8^+^ T cells (red gate). (**b**) Cytokine production by HCV epitope-specific CD8^+^ T cells of five patients with HCV infection (HCV; six HCV epitope-specific CD8^+^ T-cell responses), seven spontaneous resolvers (SpR; ten HCV-epitope-specific CD8^+^ T-cell responses) and five FLU epitope-specific CD8+ T-cell responses (FLU, *n*=5). (**c**–**e**) *In vitro* peptide-expanded HCV epitope-specific CD8^+^ T cells from HCV-infected patients (*n*=9; 12 HCV epitope-specific CD8^+^ T-cell responses) or spontaneous resolvers (*n*=8; 13 HCV epitope-specific CD8^+^ T-cell responses) were analysed for cytokine production after 5 h of peptide-specific re-stimulation. (**c**) Representative dot plots showing cytokine production by *in vitro* HCV peptide-expanded CD8^+^ T cells after HCV peptide-specific re-stimulation (gated on bulk CD8^+^ T cells). Percentages of the respective quadrant are depicted. (**d**) Frequencies of IFNγ- (left) and TNF-producing (right) *in vitro* peptide-expanded HCV epitope-specific CD8^+^ T cells are shown in the statistical graphs. (**e**) TNF production by IFNγ^+^ *in vitro* peptide-expanded CD8^+^ T cells in per cent. Bar charts show the median value with interquartile range. Statistical significance was assessed by Friedman test (HCV/DAA) and by unpaired Kruskal−Wallis (HCV, SpR, FLU). Multiple comparisons were performed with Dunn's test. (*P<0.05.)

In chronic infection, HCV-specific CD8^+^ T cells display impaired cytokine secretion and consequently diminished antiviral efficacy due to their functional exhaustion^1^,^36^,^37^. This is in contrast to memory HCV-specific CD8^+^ T cells after spontaneous virus elimination that exhibit potent antiviral efficacy^1^,^36^. To assess the capability of memory-like HCV epitope-specific CD8^+^ T cells that are maintained after DAA-mediated HCV elimination to produce cytokines, we performed a combined peptide/MHCI tetramer and intracellular cytokine-staining assay. As shown in Fig. 5a,b, peptide stimulation did not induce *ex vivo* production of IFNγ or TNF by HCV epitope-specific CD8^+^ T cells that persisted following DAA therapy.

In contrast, FLU epitope-specific CD8^+^ T cells were able to produce IFNγ and TNF after FLU M1_58_ peptide stimulation (Fig. 5b and Supplementary Fig. 11). Of note, we also could not detect *ex vivo* cytokine production after peptide stimulation of HCV epitope-specific CD8^+^ T cells derived from donors that spontaneously eliminated HCV possibly reflecting reduced assay sensitivity due to the combination of peptide/MHCI tetramer staining and peptide stimulation (Fig. 5b and Supplementary Fig. 12).

Next, we determined the capability of HCV epitope-specific CD8^+^ T cells to produce cytokines after HCV peptide-specific expansion of CD8^+^ T cells. Importantly, we could detect cytokine production by HCV epitope-specific CD8^+^ T cells after a 14-day expansion (Fig. 5c,d). Interestingly, peptide-expanded HCV-specific CD8^+^ T cells derived from donors at the end of DAA therapy exhibited increased IFNγ production (Mdn: 1.13%; IQR: 0.23–1.86%) compared to therapy baseline (Mdn: 0.14%; IQR: 0–0.53%) (Fig. 5d). To dissect cytokine production from proliferation, we analysed TNF production by IFNγ+ expanded HCV epitope-specific CD8^+^ T cells (Fig. 5e). Strikingly, TNF production was elevated among IFNγ+ HCV epitope-specific CD8^+^ T cells after DAA therapy (Baseline: Mdn: 2.88%; IQR: 0.39–8.52%; end of therapy (EOT): Mdn: 6.39%; IQR: 4.05–23.22%). However, TNF production was still decreased compared to IFNγ+ HCV epitope-specific CD8^+^ T cells present after spontaneous viral elimination (Mdn: 32.10%; IQR: 16.15–54.32%) suggesting reduced poly-functionality of HCV epitope-specific CD8^+^ T cells present after DAA-mediated viral elimination as compared to spontaneous HCV resolution.

### TCF1 expression correlates with proliferative capacity

**Figure 6:**
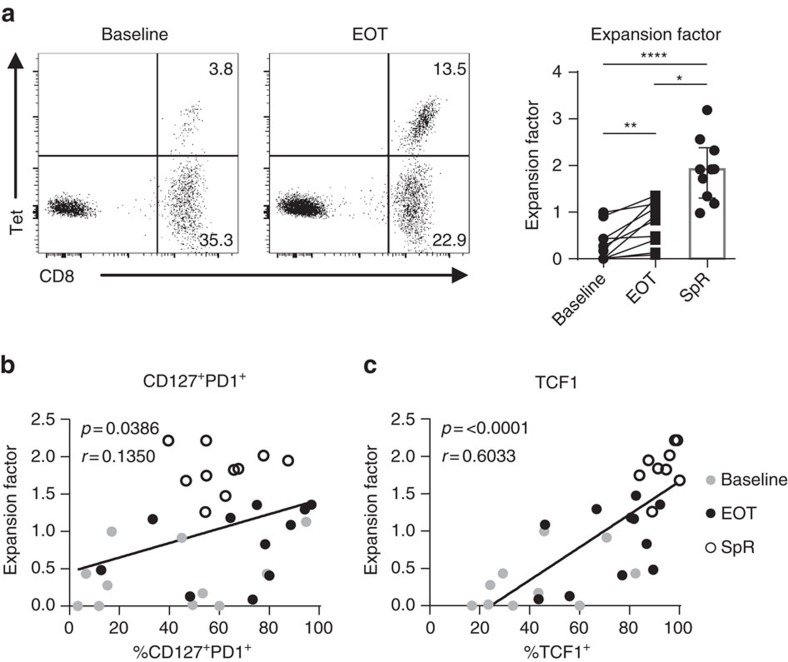
Proliferative capacity of HCV-specific CD8^+^ T cells correlates with TCF1 expression. HCV epitope-specific CD8^+^ T cells of eight HCV-infected patients (eleven HCV epitope-specific CD8^+^ T-cell responses) and four spontaneous resolvers (SpR; five HCV epitope-specific CD8^+^ T-cell responses) were expanded at baseline and at end of therapy (EOT) for 14 days by HCV epitope-specific peptides and proliferative capacity was determined by the expansion factor. The expansion factor is the logarithmic fold-increase in absolute numbers of HCV epitope-specific CD8^+^ T cells from day 0 to day 14 of *in vitro* culture. (**a**) Representative dot plots of HCV epitope-specific CD8^+^ T cells determined by peptide/MHCI and CD8 staining at day 14 of expansion at baseline (left plot) or at end of therapy (EOT; right plot), respectively. Quantification of proliferative capacity of HCV epitope-specific CD8^+^ T cells derived from donors at baseline, at EOT and of SpR is depicted on the right. (**b**,**c**) Correlation analyses of the expansion factor with the abundance of memory-like CD127^+^PD1^+^ (**b**) or TCF1^+^ (**c**) HCV epitope-specific CD8^+^ T cells at day 0 of the assay, respectively (grey: baseline; black: EOT; open circles: SpR). Bar chart shows the median value with interquartile range. Statistical significance was assessed with Wilcoxon test (HCV/DAA Baseline to EOT), multiple comparisons with Kruskal–Wallis and Dunn's test (SpR to cHCV Baseline/EOT). Pearson correlation in (**b**,**c**) was performed. (*P<0.05; **P<0.01; ****P<0.0001.)

A further aspect of T-cell functionality is the proliferative capacity upon antigen encounter. Therefore, we tested HCV epitope-specific CD8^+^ T-cell proliferation before and after DAA-mediated HCV elimination. For this, we determined the fold expansion of HCV epitope-specific CD8^+^ T cells after 14-day HCV peptide-specific expansion assays. As previously shown^38^, the majority of analysed patients showed a significant increase in the expansion of HCV epitope-specific CD8^+^ T cells after antigen removal (Fig. 6a). However, the proliferative capacity of HCV epitope-specific CD8^+^ T cells obtained at the end of DAA treatment (expansion factor: Mdn: 0.63; IQR: 0.13–1.16) was reduced compared to HCV epitope-specific CD8^+^ T cells obtained from patients with spontaneous viral clearance (expansion factor: Mdn: 2.32; IQR: 1.63–2.87). We next addressed the question whether the abundance of CD127^+^PD1^+^ HCV epitope-specific CD8^+^ T cells accounts for the proliferative potential of the HCV epitope-specific CD8^+^ T-cell population. Indeed, the proliferative capacity of HCV epitope-specific CD8^+^ T-cell populations correlated with the abundance of CD127^+^PD1^+^ and particularly with that of TCF1^+^ HCV epitope-specific CD8^+^ T cells (Fig. 6b,c). These results suggest that TCF1 defines the proliferative capacity of CD127^+^PD1^+^ virus-specific CD8^+^ T cells during and after chronic antigen stimulation.

### Memory-like T cells provide recall response

**Figure 7:**
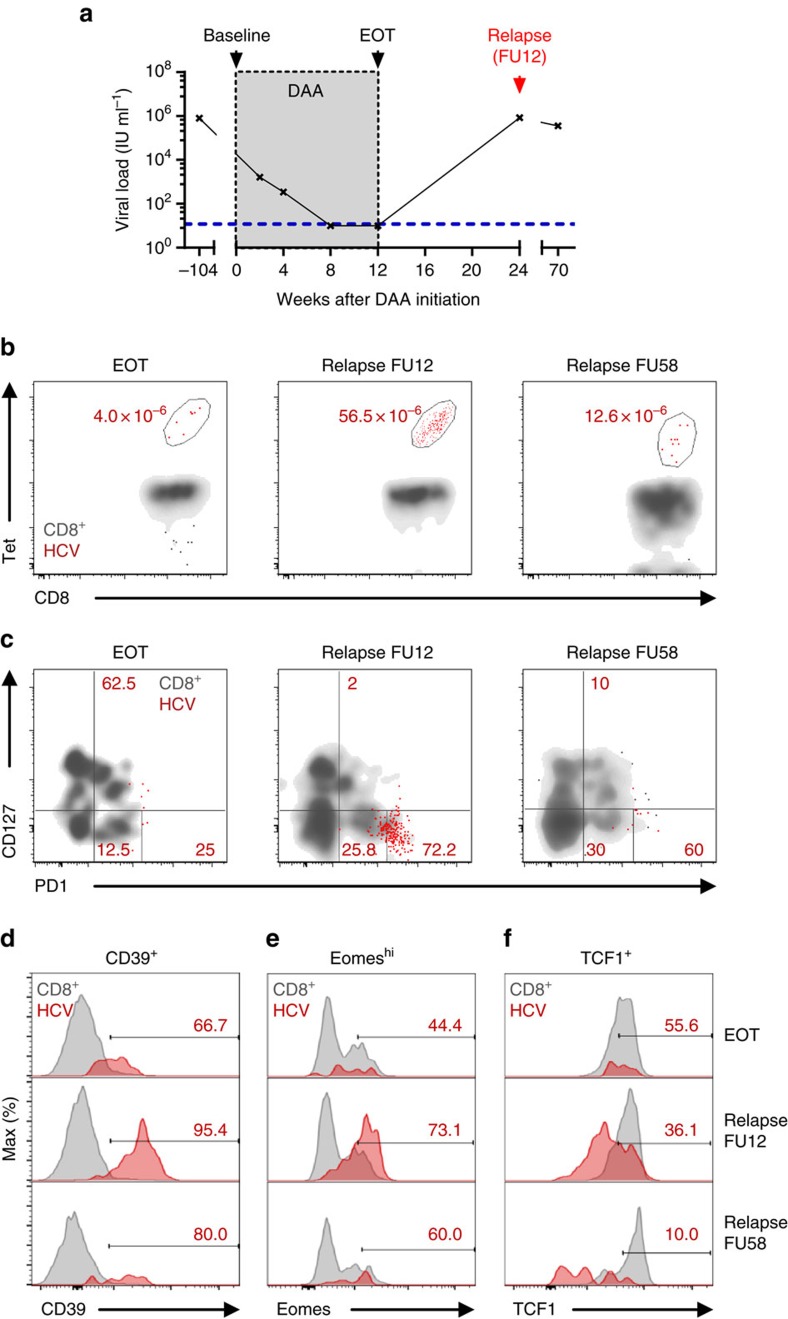
Memory-like HCV-specific CD8^+^ T cells provide recall response after viral relapse. Longitudinal analyses of HCV epitope-specific CD8^+^ T cells of one patient with HCV relapse after DAA-mediated antigen elimination. (**a**) Viral loads (HCV RNA) in serum samples at the indicated time-points were determined. Lower detection limit is indicated by blue dashed line. (**b**) Frequencies of NS3_1073_-specific CD8^+^ T-cell populations at end of therapy (EOT) and after viral relapse at FU12 and FU58 are shown. (**c**) CD127/PD1 subset distribution and relative expression of CD39 (**d**), Eomes^hi^ (**e**) and TCF1 (**f**) of NS3_1073_-specific CD8^+^ T cells were assessed at the indicated time-points (red: HCV epitope-specific CD8^+^ T cells; grey: corresponding CD8^+^ T cells).

We had the single opportunity to analyse the re-expansion capacity of memory-like TCF1^+^CD127^+^PD1^+^ HCV epitope-specific CD8^+^ T cells *in vivo*, since one DAA-treated patient presented with viral relapse at the 12-week follow-up (FU12) visit (Table 1 and Fig. 7a). Importantly, as shown in Fig. 7b, viral relapse and thus antigen re-exposure led to a vigorous expansion of HCV NS3_1073_-specific CD8^+^ T cells that was accompanied with CD127/PD1 subset re-distribution (Fig. 7c and Supplementary Fig. 13) and concomitant phenotypic changes (Fig. 7d–f). More specifically, viral relapse led to a 2.9-fold increase of CD127-PD1^hi^ HCV NS3_1073_-specific T cells that were roughly maintained at a 1-year follow-up visit (2.4-fold increase relative to EOT). In concert with this, expression of CD39 and Eomes increased (Fig. 7d,e) while expression of TCF1 decreased (Fig. 7f) indicative of the re-generation of terminally exhausted effector subsets. These results, therefore, clearly suggest that memory-like TCF1^+^CD127^+^PD1^+^ HCV epitope-specific CD8^+^ T cells are able to re-expand efficiently in response to antigen re-exposure and can give rise to terminally exhausted effector subsets characterized by TCF1-CD127-PD1^hi^ expression in the context of viral relapse.

## Discussion

In this study, we analysed circulating HCV epitope-specific CD8^+^ T cells in a clinically relevant model. We could define key characteristics of distinct heterogeneous HCV epitope-specific CD8^+^ T-cell subsets during antigen persistence and elimination in the peripheral blood. Based on CD127/PD1 co-expression analyses, we found that CD127^+^PD1^+^, CD127-PD1^lo^ and CD127-PD1^hi^ subsets contribute to the HCV epitope-specific CD8^+^ T-cell pool during antigen persistence. Intriguingly, however, only the CD127^+^PD1^+^ HCV epitope-specific CD8^+^ T-cell subset is maintained after DAA-mediated HCV clearance indicating memory-like survival characteristics. The CD127^+^PD1^+^ HCV-specific CD8^+^ T-cell subset is characterized by a high expression of the transcription factor TCF1 that has been shown to be essential for the establishment and persistence of CD8^+^ T-cell responses^25^,^27^,^32^,^33^,^39^. More specifically, it has been reported that TCF1 defines the proliferative capacity of virus-specific CD8^+^ T cells in the LCMV mouse model of acute and chronic infection^25^,^27^,^40^. In line with this, we could correlate the *in vitro* proliferative capacity of HCV epitope-specific CD8^+^ T cells with TCF1 expression clearly suggesting that the TCF1 expressing subset contains the proliferative capacity within the HCV epitope-specific CD8^+^ T-cell pool. This was further supported by two *in vivo* observations. First, an increased proliferative capacity of HCV epitope-specific CD8^+^ T cells after cessation of chronic antigen stimulation occurred in parallel with the increase of the TCF1^+^CD127^+^PD1^+^ HCV epitope-specific T-cell subset. Second, TCF1^+^CD127^+^PD1^+^ HCV epitope-specific CD8^+^ T cells robustly expanded in a patient with viral relapse. This capacity of robust secondary expansion represents a hallmark of CD8^+^ T-cell memory clearly supporting the memory-like character of this HCV-specific CD8^+^ T-cell subset. Consistently, a recent study in the mouse model of chronic LCMV infection has shown that TCF1^+^ virus-specific CD8^+^ T cells contain recall proliferative capacity after transfer in acutely infected mice^27^. Hence, in humans and mice, antigen persistence does not completely abrogate memory potential regarding survival and proliferation characteristics of virus-specific CD8^+^ T-cell pools. Of note, in human HCV infection, this holds true after years of persistent antigen exposure at least in the peripheral blood broadening the prospects for therapeutic interventions.

Remarkably, memory-like TCF1^+^CD127^+^PD1^+^ HCV epitope-specific CD8^+^ T cells present after DAA-mediated HCV elimination do not fully resemble memory CD8^+^ T cells present after spontaneous viral clearance. Although they share phenotypic and molecular memory properties, like CD127, BCL2 and TCF1 expression, memory-like HCV-specific CD8^+^ T cells display a higher expression of PD1 and Eomes indicative of T-cell exhaustion. This fits to the unique transcriptome of TCF1^+^ LCMV-specific CD8^+^ T cells in chronically infected mice that includes key factors of both signatures, T-cell memory and exhaustion^25^,^27^. Furthermore, TCF1^+^ virus-specific CD8^+^ T cells from chronic LCMV infection also maintained an exhausted cytokine profile after re-expansion *in vivo*^27^. Similarly, memory-like HCV-specific CD8^+^ T cells also revealed impaired cytokine production after DAA-mediated compared to spontaneous antigen elimination. These cells therefore exhibit phenotypic and functional characteristics of T-cell memory and exhaustion. Hence, memory-like HCV-specific CD8^+^ T cells and TCF1^+^ LCMV-specific CD8^+^ T cells, both induced during chronic infections, are clearly distinct from memory CD8^+^ T cells generated in self-limiting infections suggesting a divergent T-cell differentiation program. While conventional memory T cells are equipped with extensive polyfunctionality to rapidly clear re-infection, memory-like T cells established during chronic infection might acquire a differentiation program that is adjusted to the setting of persistent antigen allowing pathogen control without immunopathology. This might have important implications for vaccination strategies aiming to boost virus-specific CD8^+^ T-cell responses during and after chronic antigen stimulation.

Noteworthy, memory-like TCF1^+^ LCMV-specific CD8^+^ T cells from chronically infected mice were able to control LCMV infection after transfer in acutely infected mice^27^. In humans, however, it is unclear whether memory-like TCF1^+^CD127^+^PD1^+^ HCV-specific CD8^+^ T cells that persist after DAA-mediated HCV clearance will be able to provide protection against re-infection. In the chimpanzee model of chronic HCV infection, a recent study has suggested that exhausted HCV-specific CD8^+^ T-cell populations do not functionally recover after DAA-mediated cure and are unable to prevent virus persistence after re-infection^41^,^42^. In line with this, multiple re-infections of DAA-treated patients have been reported also questioning the presence of protective immune memory after DAA clearance although the role of genotypes and viral escape has not been addressed. Clearly, additional studies are needed to address the protective potential of memory-like HCV-specific CD8^+^ T cells. Our finding, however, of a significant re-expansion of this HCV-specific CD8^+^ T-cell subset combined with a decent production of cytokines indicates that protective immunity may be possible at least in a subset of subjects.

Importantly, recall expansion of memory-like TCF1^+^CD127^+^PD1^+^ HCV-specific CD8^+^ T cells was accompanied by re-occurrence of the TCF1-CD127-PD1^hi^ subset after re-exposure to persisting antigen suggesting a progenitor-progeny relationship of these two subsets during chronic antigen recognition. The CD127-PD1^hi^ subset of HCV epitope-specific CD8^+^ T cells highly expressed inhibitory receptors and CD39 and exhibited the transcriptional Tbet^dim^Eomes^hi^ signature that is characteristic for terminal exhaustion^6^,^12^,^15^. Of note, it has previously been shown that intrahepatic HCV-specific CD8^+^ T cells obtained from chronically HCV-infected patients exclusively displayed a Tbet^dim^Eomes^high^ profile^15^ raising the question of the CD127/PD1 subset distribution at the site of HCV persistence. Furthermore, CD127-PD1^hi^ HCV epitope-specific CD8^+^ T cells also expressed perforin, at least to some extent, and increased levels of CD122 indicative of effector cell differentiation^34^,^43^,^44^,^45^. Thus, the TCF1-CD127-PD1^hi^ subset of HCV-specific CD8^+^ T cells contains terminally exhausted effector cells. Importantly, this terminally exhausted TCF1-CD127-PD1^hi^ effector subset was absent or disappeared from the HCV epitope-specific CD8^+^ T-cell population when the corresponding viral epitope was removed either naturally in the context of viral escape mutations or therapeutically by DAA therapy. In sum, these results clearly indicate that terminal exhaustion of HCV epitope-specific CD8^+^ T cells is linked to ongoing antigen recognition and support previous findings from the LCMV model showing antigen dependence of terminal exhausted virus-specific CD8^+^ T cells^46^,^47^,^48^. The mechanism responsible for the disappearance of the TCF1-CD127-PD1^hi^ HCV epitope-specific CD8^+^ T cells after antigen elimination is not entirely clear but may be linked to a reduced lifespan of these cells due to terminal differentiation. In agreement with this hypothesis, we found low expression of the pro-survival molecule BCL-2 that has been associated with a higher apoptosis rate of virus-specific CD8^+^ T cells^49^. Furthermore, the lack of CD127 expression on the TCF1-CD127-PD1^hi^ subset of HCV epitope-specific CD8^+^ T cells abrogates sensing of IL-7 that is required for antigen-independent survival of CD8^+^ T cells^50^. Persisting antigen is therefore required to drive the generation of terminal exhausted HCV-specific CD8^+^ T cells possibly arising from less differentiated memory-like subsets similar to progenitor and terminal subsets of virus-specific CD8^+^ T cells found in the mouse model of chronic LCMV infection^15^,^25^,^26^,^27^. Since both subsets are required to sustain the virus-specific CD8^+^ T-cell response during antigen persistence in mice it is tempting to speculate that a similar mechanism also exists in chronic human infection.

In conclusion, we demonstrate for the first time the emergence of a less differentiated circulating memory-like subset of virus-specific CD8^+^ T cells during antigen persistence in a relevant chronic human infection that is maintained after cessation of chronic antigen stimulation. Thus, our results give clinical significant insights into virus-specific CD8^+^ T-cell immunity after elimination of chronic viral infection and have implications for re-infection and therapeutic vaccination. In particular, memory-like HCV epitope-specific CD8^+^ T cells contain the proliferative potential and provide recall responses including re-expansion of HCV epitope-specific CD8^+^ T cells and re-occurrence of terminally exhausted subsets. Thus, this memory-like subset appears to be central to maintain virus-specific CD8^+^ T-cell responses during chronic infection and after consequent DAA-mediated viral clearance rendering this subset of virus-specific CD8^+^ T cells a promising target to boost CD8^+^ T-cell responses in immune therapeutic interventions.

## Methods

### Study cohort

Patients were recruited at the Department of Medicine II of the University Hospital Freiburg, Germany. 35 HLA-A*02 positive patients with chronic HCV infection were included in this study. Twenty nine of these patients could be followed through IFNα-free DAA therapy and five patients were treated with IFNα-based regiments. All patients included in the study were infected with HCV genotype 1a or 1b. Viral loads were determined as part of the clinical diagnostics at the University Hospital Freiburg. In addition, 15 HLA-A*02 positive patients who spontaneously resolved HCV infection (anti-HCV positive, HCV-RNA negative) were included. Confirmation of HLA-A*02 was performed by antibody staining and four-digit HLA-typing by next generation sequencing. Characteristics of the patient cohort can be found in Table 1.

Written informed consent was obtained in all cases and the study was conducted according to federal guidelines, local ethics committee regulations (Albert-Ludwigs-Universität, Freiburg, Germany) and the Declaration of Helsinki (1975).

### PBMC isolation

PBMCs were isolated from EDTA anticoagulated blood patient samples through Pancoll (Pan-Biotech) density gradient centrfugation. For best data comparability, all PBMC samples from one patient during therapy were frozen and thawed simultaneously at the day of experiment. PBMCs were thawed in complete medium (RPMI 1640 with 10% fetal bovine serum, 1% penicillin-streptomycin and 1.5% 1 M HEPES (all Thermo Fisher, Germany)) and incubated for 15–30 min at 37 °C in complete medium containing 50 U ml^−1^ benzonase (Sigma, Germany) before processing.

### Peptides and tetramers

Peptides of HLA-A*02-restricted HCV-derived epitopes ((I) genotype 1a: NS3_1073_, CINGVCWTV; NS3_1406_, KLVALGINAV; NS5_2594_, ALYDVVTKL; (II) genotype 1b: NS3_1073_, CVNGVCWTV; NS3_1406_, KLSGLGLNAV; NS5_2594_, ALYDVVSTL), variant epitopes matching patient viral sequences (Table 1), cytomegalovirus (CMV)-derived epitope pp65_495_, NLVPMVATV, influenza virus (FLU)-derived epitope M1_58_, GILGFVFTL, and Epstein—Barr Virus (EBV)-derived epitope BMFL1_280_, GLCTLVAML, were obtained from Genaxxon, Germany. Peptides were dissolved in dimethyl sulfoxide (Sigma, Germany) at 20 mg ml^−1^ and diluted in complete medium to 1 mg ml^−1^ before usage. Major histocompatibility complex (MHC) class I epitope-specific tetramers were generated by conjugation of biotinylated peptide-MHC class I monomers (kind gift from David Price, Cardiff University) with PE- or APC-conjugated streptavidin at a MHCI:Strepatividin molar ratio of 5:1.

### Tetramer enrichment

Tetramer enrichment was performed as previously described^51^. Briefly, PBMCs were labelled with peptide/HLA-A*02 tetramers coupled to either phycoerythrin (PE) or allophycocyanin (APC). Subsequent enrichment was performed with anti-PE/APC beads applying MACS technology (Miltenyi Biotec, Germany) according to the manufacturer's protocol. Enriched virus-specific CD8^+^ T cells were used for flow cytometry or *ex vivo* stimulation assays. To restrict analysis only to those cells that actually encountered antigen before, CD45RA^+^CCR7^+^ or CD45RA^+^CD27^+^ naive virus-specific CD8^+^ T cells were excluded (Supplementary Fig. 2). Samples were excluded when <5 cells could be detected after peptide/HLA-A*02 tetramer-based enrichment. Frequencies of virus-specific CD8^+^ T cells were calculated as described by Alanio *et al*.^51^.

### Multiparametric flow cytometry

The following reagents were used for multi-parametric flow cytometry: anti-HLA-A*02 (BB7.2, 1:100), anti-HLA-B*27 (FD705-9E1E10, 1:100), anti-CD8 (SK1, 1:50), anti-Eomes (WD1928, 1:30), anti-T-bet (4B10, 1:30), anti-CD14 (61D3, 1:100), anti-CD19 (HIB19, 1:100), anti-TIGIT (MBSA43, 1:30) (eBioscience, Germany). Anti-CCR7 (G043H7, 1:30), anti-CCR7 (G043H7, 1:30), anti-CD127 (A019D5, 1:30), anti-CD45RA (HI100, 1:200), anti-PD1 (EH12.2H7, 1:30), anti-TCF1 (C63D9, 1:30), anti-CD8 (HIT8a, 1:50), anti-2B4 (C1.7, 1:100), anti-CCR7 (150503, 1:30), anti-Rabbit IgG (Poly4064, 1:200), anti-CD45RA (HI100, 1:200), anti-Perforin (dG9, 1:50), anti-CD122 (TU27, 1:30), anti-IFN-γ (4S.B3, 1:50) (BioLegend, UK). Anti-IFN-γ (25723.11, 1:30), anti-Bcl-2 (Bcl-2/100, 1:100), anti-TNF (MAb11, 1:50), anti-CD39 (TU66, 1:30), anti-CD8 (RPA-T8, 1:100), anti-CD14 (MϕP9, 1:100), anti-CD19 (SJ25C1, 1:100) (BD Biosciences, Germany). Anti-TCF1 (C63D9, 1:100) (Cell signaling, Germany). Fixable Viability Dyes (eFluor506 (1:100) or eFluor780 (1:5000), eBioscience, Germany) and 7-AAD (1:30) (BD Biosciences, Germany) were used for live/dead discrimination. FoxP3/Transcription Factor Staining Buffer Set (eBioscience, Germany) was applied according to the manufacturer's instructions to stain for cytoplasmic and nuclear molecules. Cells were fixed with paraformaldehyde (2% PFA) before sample acquisition on a FACSCanto II or an LSRFortessa (BD Biosciences, Germany).

### T-cell expansion and calculation of expansion factor

Around 1–2 × 10^6^ PBMCs were stimulated with epitope-specific peptides (10 μg ml^−1^) and anti-CD28 (clone CD28.2, 0.5 μg ml^−1^, BD Biosciences, Germany) in 1 ml complete medium and incubated at 37 °C for 14 days. At day 3, 7 and 10, culture was supplemented with 0.5 ml of fresh medium including 20 IU ml^−1^ rIL-2 (Miltenyi Biotec, Germany). Tetramer and intracellular cytokine staining were performed at day 14. The expansion index was calculated as follows: [A] The absolute number of virus-specific CD8^+^ T cells added at day 0 of *in vitro* expansion was calculated based on peptide/MHCI tetramer enrichments (see above). [B] At day 14 of *in vitro* expansion, the absolute number of expanded virus-specific CD8^+^ T cells was determined based on direct FACS analyses. The expansion index was then calculated as (([B]/[A])+1), allowing subsequent logarithmic calculation despite zero values, resulting in the expansion factor=log(expansion index).

### Cytokine production

Cytokine production of virus-specific CD8^+^ T cells after 14 days of *in vitro* expansion or directly *ex vivo* after peptide/MHCI tetramer enrichment of virus-specific CD8^+^ T cells was induced by re-stimulating cells with epitope-specific peptides (10 μg ml^−1^) in the presence of brefeldin A (GolgiPlug; 0.5 μl ml^−1^) and monensin (GolgiStop; 0.325 μl ml^−1^) (BD Biosciences, Germany) for 5 h at 37 °C. Stimulation with phorbol 12-myristate 13-acetate (PMA; 50 ng ml^−1^, Sigma, Germany) and Ionomycin (1 μg ml^−1^, Sigma, Germany) was performed as positive control. Subsequently, cells were stained as described above.

### Viral sequencing

RNA was extracted from sera using the QIAmp viral RNA minikit (Qiagen, Germany) followed by reverse transcription (RT; SuperScript III First-Strand Kit, Invitrogen, Germany) and DNA amplification by nested PCR (both PCRs via GoTaq G2 Flexi DNA Polymerase Kit, Promega, Germany) according to manufacturers' protocols. Specific primers for HCV genotype 1a and 1b can be found in Supplementary Table 3.

### T-cell cross-reactivity with viral sequence variations

Epitope-specific T-cell lines were generated from PBMCs by stimulation with viral peptides (wildtype sequences) and expansion for 14 days as described above. On day 14, T-cell lines were tested for IFNγ production upon peptide-specific stimulation as described above. For stimulation, HLA-A*02:01 positive EBV-transformed B-LCLs were loaded with peptides ((a) wildtype peptide; (b) viral sequence variation peptide; (10 μg ml^−1^)) overnight, then washed extensively and finally co-cultured with T-cell lines at an E:T ratio of 1:1. For production of EBV-transformed B-LCLs from HLA-A*02 positive subjects, supernatant from the EBV-producing B95-8 marmoset lymphoblastoid cell line was incubated with donor PBMCs of patients with known HLA-A*02 genotype followed by cyclosporine treatment^52^.

Cross-recognition definitions were based on Cox *et al*.^53^. Briefly, loss of recognition was defined as at least 20-fold reduction in IFNγ production upon variant peptide stimulation compared to wildtype peptide stimulation; 2-fold and less reduction in IFNγ production was defined as cross-recognition.

### Statistics

Flow cytometry data were analysed using FlowJo software version 9 and 10 (Treestar, USA). During data analysis by FlowJo, no further sub-gate analysis was performed when <5 cells were detected. Statistical analysis was performed with GraphPad 6 software (GraphPad Prism Software Inc., USA). Bar charts show median values with interquartile range. Two-tailed tests were applied to a significance level of 95%. Statistical tests used are indicated in the figure legends. When patients did not appear to one single study time point for unknown reasons, missing at random (MAR) was assumed for statistical analysis. Paired analysis of these data sets was then performed by applying the lastly collected value to the missing time point. (*P<0.05; **P<0.01; ***P<0.001; ****P<0.0001.)

### Data availability

The data that support the findings of this study are available from the corresponding author on request.

## Additional information

**How to cite this article:** Wieland, D. *et al*. TCF1^+^ hepatitis C virus-specific CD8^+^ T cells are maintained after cessation of chronic antigen stimulation. *Nat. Commun.* **8,** 15050 doi: 10.1038/ncomms15050 (2017).

**Publisher's note:** Springer Nature remains neutral with regard to jurisdictional claims in published maps and institutional affiliations.

## Supplementary Material

Supplementary Information Supplementary Figures and Supplementary Tables

Peer Review File

## Figures and Tables

**Table 1:** Patient characteristics.

**Pt**	**D**	**Cohort**	**Treatment**	**Outcome**	**gt**	**NS3**_**1073**_	**NS3**_**1406**_	**NS5**_**2594**_	**VL**	**Age**	**Sex**	**CIR**
1	cHCV	DAA	Ledipasvir/Sofosbuvir 8 wk	SVR	1a		------V---		0.15	47	f	no
2	cHCV	DAA	Ledipasvir/Sofosbuvir 12 wk	SVR	1a	---------			0.26	60	m	no
3	cHCV	DAA	Ledipasvir/Sofosbuvir 12 wk	SVR	1a	---------	----------		1.06	52	m	no
4	cHCV	DAA	Ledipasvir/Sofosbuvir 12 wk	SVR	1a		------V---		3.20	66	f	no
5	cHCV	DAA	Ledipasvir/Sofosbuvir 12 wk	SVR	1a	---------		----------	0.63	59	f	no
6	cHCV	DAA	Ledipasvir/Sofosbuvir 12 wk	SVR	1a		----------	----------	0.68	64	f	no
7	cHCV	DAA	Ledipasvir/Sofosbuvir 12 wk	SVR	1a	---------		----------	2.96	50	m	no
8	cHCV	DAA	Ledipasvir/Sofosbuvir 12 wk	SVR	1a	---------			ND	49	m	no
9	cHCV	DAA	Ledipasvir/Sofosbuvir 12 wk	SVR	1a	---------		----------	15.0	58	m	no
10	cHCV	DAA	Ledipasvir/Sofosbuvir 12 wk	SVR	1a	---------			0.29	59	m	no
11	cHCV	DAA	Ledipasvir/Sofosbuvir 12 wk	SVR	1a	---------			1.03	47	m	no
12	cHCV	DAA	Ledipasvir/Sofosbuvir/Ribavirin 12 wk	SVR	1a		------V---		0.04	73	f	no
13	cHCV	DAA	Ledipasvir/Sofosbuvir/Ribavirin 12 wk; untreated after relapse	RELAPSE	1a	---------			ND	62	m	yes
14	cHCV	DAA	Ledipasvir/Sofosbuvir 8 wk	SVR	1b	-I-------			0.70	49	m	no
15	cHCV	DAA	Ledipasvir/Sofosbuvir 8 wk	SVR	1b	---------			0.30	60	f	no
16	cHCV	DAA	Ledipasvir/Sofosbuvir 12 wk	SVR	1b	---------			5.30	78	f	no
17	cHCV	DAA	Ledipasvir/Sofosbuvir 12 wk	SVR	1b	---------			1.00	63	f	no
18	cHCV	DAA	Ombitasvir/Paritaprevir/Ritonavir/Dasabuvir 12 wk	SVR	1b	-I-------			1.23	29	m	no
19	cHCV	DAA	Ombitasvir/Paritaprevir/Ritonavir/Dasabuvir/Ribavirin 12 wk	SVR	1b	---------			0.04	66	f	no
20	cHCV	DAA	Ledipasvir/Sofosbuvir/Ribavirin 12 wk	SVR	1b	-I-------			0.61	58	f	no
21	cHCV	untreated	-	-	1a	---------			1.36	41	m	no
22	cHCV	untreated	-	-	1a	---------			4.59	51	m	no
23	cHCV	untreated	-	-	1a		----------		0.41	55	f	no
1	cHCV	ESC	Ledipasvir/Sofosbuvir 8 wk	SVR	1a	--------A			0.15	47	f	no
4	cHCV	ESC	Ledipasvir/Sofosbuvir 12 wk	SVR	1a	--------A			3.2	66	f	no
12	cHCV	ESC	Ledipasvir/Sofosbuvir/Ribavirin12 wk	SVR	1a	--------A			0.04	73	f	no
24	cHCV	ESC	Ledipasvir/Sofosbuvir 12 wk	SVR	1b	--S------			2.46	56	f	no
25	cHCV	ESC	-	SVR	1b	-------SA			0.04	57	m	no

**Pt**	**D**	**Cohort**	**Approx. time after primary HCV infection**	**Age**	**Sex**	**CIR**
26	SpR	SpR	22 years	43	m	no
27	SpR	SpR	unknown	39	f	no
28	SpR	SpR	unknown	25	m	no
29	SpR	SpR	unknown	67	f	no
30	SpR	SpR	unknown	38	f	no
31	SpR	SpR	unknown	23	m	no
32	SpR	SpR	unknown	54	m	no
33	SpR	SpR	unknown	57	f	no
34	SpR	SpR	unknown	42	m	no
35	SpR	SpR	12 years	39	f	no
36	SpR	SpR	19 years	37	m	no
37	SpR	SpR	unknown	38	m	no
38	SpR	SpR	unknown	60	f	no
39	SpR	SpR	unknown	54	f	no
40	SpR	SpR	13 years	33	m	no

CIR, cirrhosis; D, diagnosis; f, female; gt, HCV genotype; m, male; ND, not defined; Pt, patient; SVR, sustained virological response; VL, baseline viral load (IU ml^−1^ × 10^6^); wk, week.

Patient characteristics are depicted. Listed are chronically HCV-infected patients (cHCV) with and without DAA treatment (DAA or untreated) and spontaneous HCV resolvers (SpR). In column NS3_1073_, NS3_1406_ and NS5_2594_ the viral sequence for each analysed CD8^+^ T-cell epitope during chronic HCV infection is shown: (-) indicates a sequence position that corresponds to wild type viral sequence, capital letters show amino acid substitutions varying from wild type sequence. Patients with analysed HCV epitope-specific CD8^+^ T cells that do not recognize viral antigen due to viral escape mutation in their corresponding epitope are listed in ESC (viral escape) cohort. If known, the approximate time after primary HCV inection of a SpR patient is indicated.
