# Supplementary material for: TCF1+ hepatitis C virus-specific CD8+ T cells are maintained after cessation of chronic antigen stimulation
Source: Nat Commun. 2017 May 3;8:15050. doi: 10.1038/ncomms15050 (PMC5418623; doi:10.1038/ncomms15050)
Supplement: Supplementary Information — Supplementary Figures and Supplementary Tables [file ncomms15050-s1.pdf]

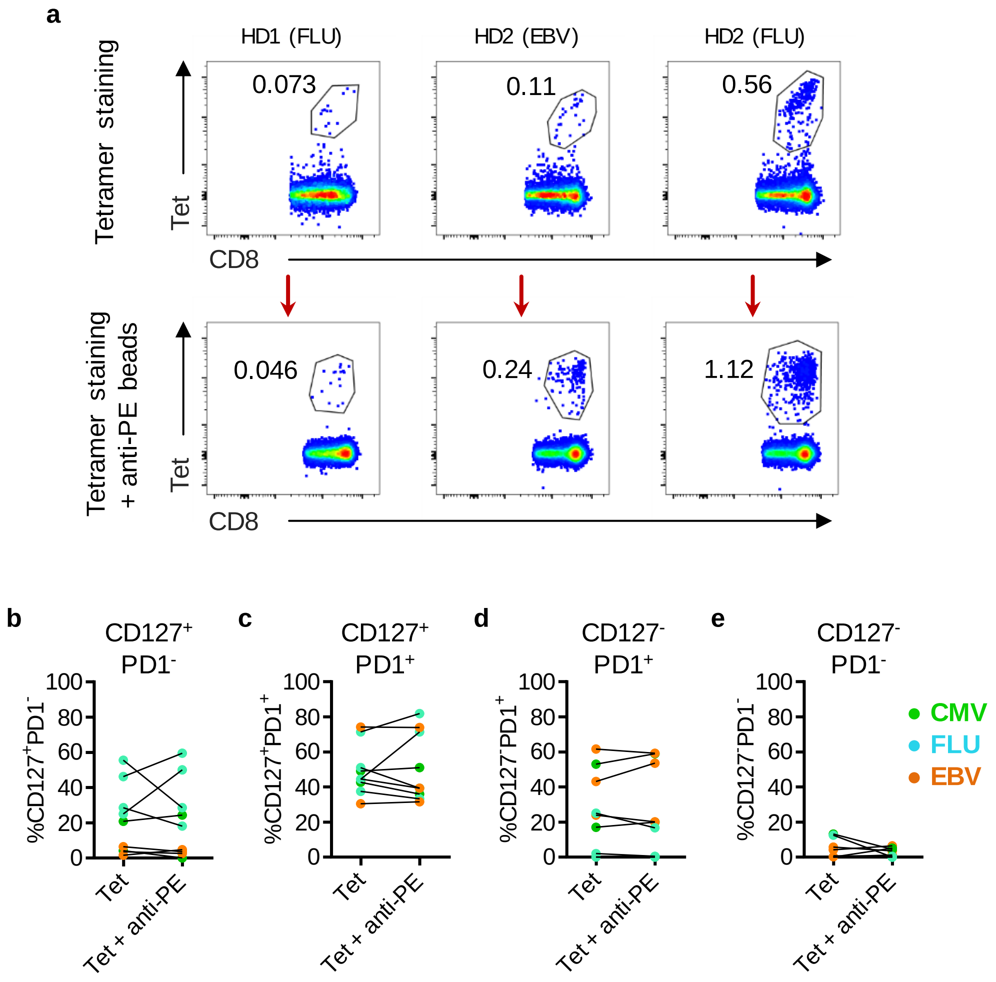

Supplementary Figure 1. Peptide/HLA-A*02 tetramer-based enrichment procedure does not enrich unspecific T cells. Peptide/MHC class I tetramer staining was performed with or without subsequent incubation of tetramer-labelled CD8+ T cells with anti-fluorochrome (anti-PE) microbeads. (a) Representative dot plots are depicted of three virus-specific CD8+ T-cell response (reaching from low to high frequency) either without anti-PE labelling (upper panel) or with labelling with anti-PE microbeads (lower panel) (2 healthy donors (HD) with 3 virus-specific CD8+ T-cell responses: 2 FLU, 1 EBV). (b-e) To exclude unspecific peptide/HLA-A*02 tetramer-labelling and thus enrichment of specific CD127/PD1 subsets, the phenotype of tetramer-labelled virus-specific CD8+ T cells was analyzed before and after co-incubation with anti-fluorochrome beads (9 epitope-specific CD8+ T-cell responses from 5 healthy donors: 4 FLU, 3 EBV, 2 CMV). Statistical significance was assessed by Wilcoxon test.

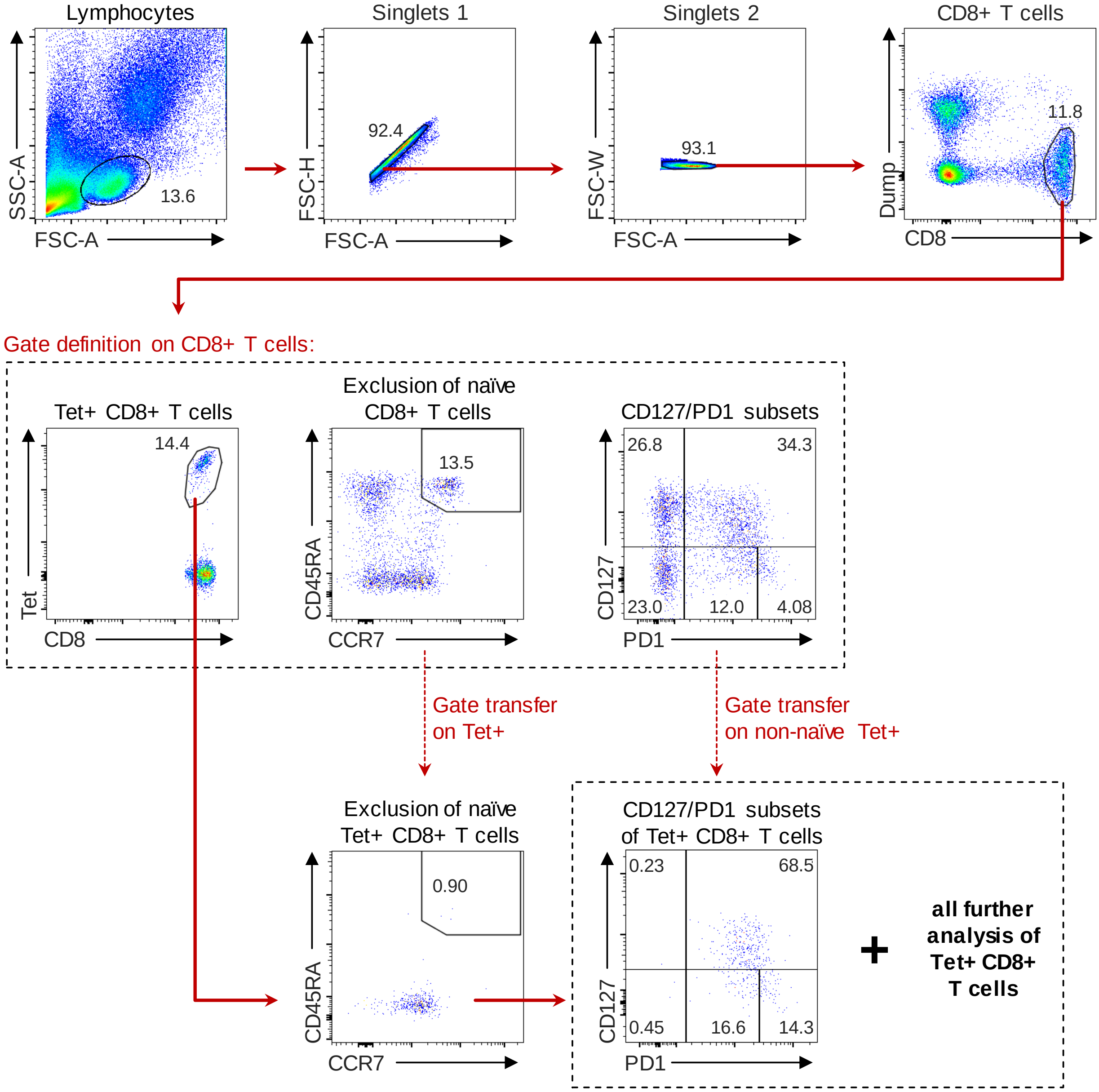

Supplementary Figure 2. Gating strategy for samples after enrichment of virus-specific T cells. After lymphocyte gating and two-way doublet exclusion, CD8+ T-cells were gated (Dump: dead cells, CD14+ cells, CD19+ cells). Bulk CD8+ T-cell gate was used to assist gating for all markers included in the sample. Naïve epitope-specific CD8+ T cells were excluded to prevent misinterpretation by non-primed T-cell populations.

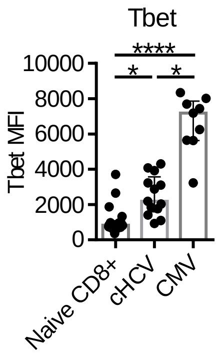

Supplementary Figure 3. HCV-specific CD8+ T cells exhibit low expression of T-bet. T-bet expression was defined in HCV epitope-specific CD8+ T cells (n=13) and compared to expression in CMV epitope-specific CD8+ T cells (n=9) and naïve CD8+ T cells (n=18) of chronically HCV-infected patients. Statistical significance was assessed by Mann-Whitney test (*, P<0.05; ****, P<0.0001.). Median with interquartile range is indicated. MFI: median fluorescence intensity.

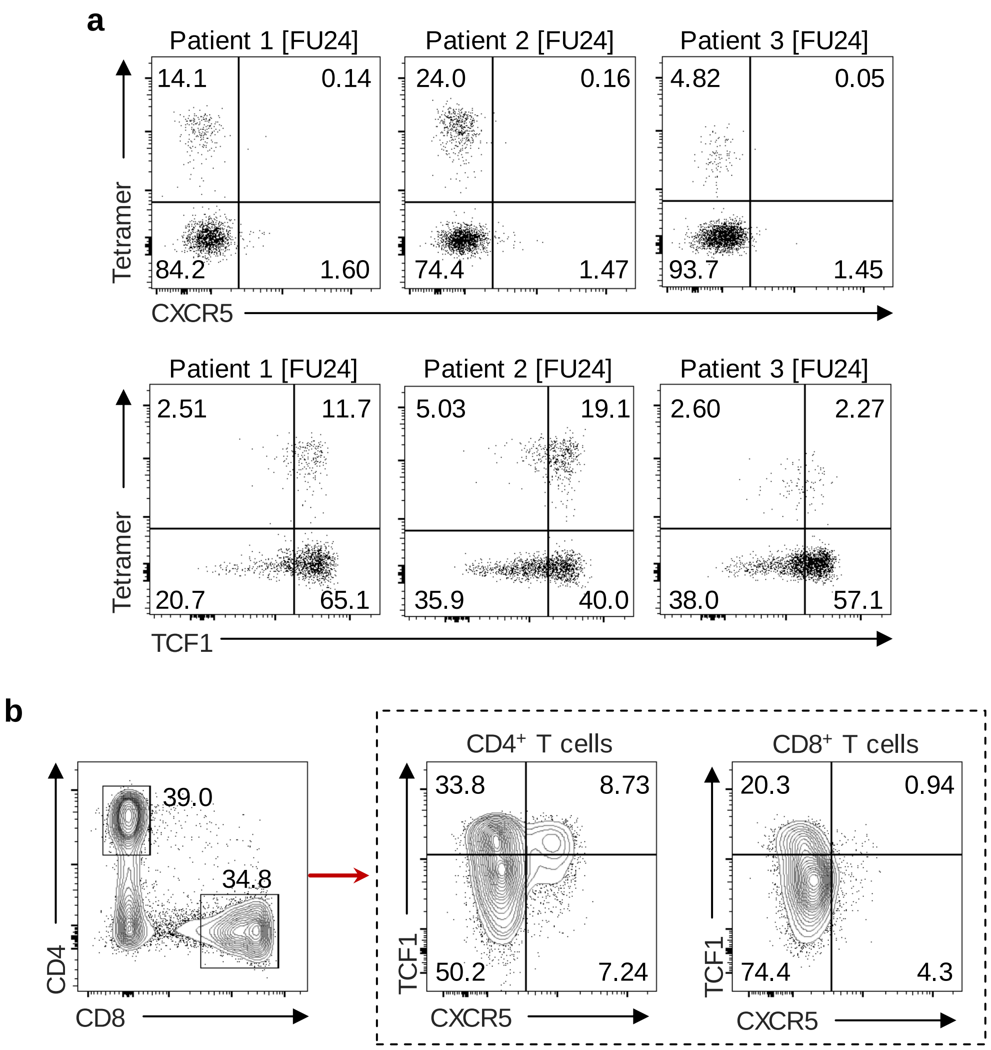

Supplementary Figure 4. PBMC-derived TCF1+ HCV-specific CD8+ T cells lack CXCR5 expression. (a) Expression of CXCR5 (upper panel) and TCF1 (lower panel) was analyzed on HCV epitope-specific CD8+ T cells 24 weeks after DAA-mediated HCV elimination (FU24). Representative data from three patients are shown. (b) Representative dot plot of PBMCs analyzed for CXCR5 and TCF1 expression on CD4+ and CD8+ T cells.

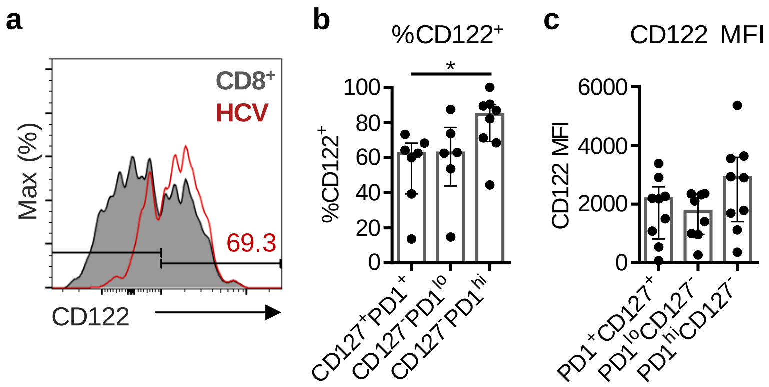

Supplementary Figure 5. CD127-PD1hi HCV-specific CD8+ T cells show high CD122 expression. (**a**) Representative flow cytometric histogram plot including gating of CD122 is depicted (red: HCV epitope-specific CD8+ T cells; grey: corresponding bulk CD8+ T cells). (**b** and **c**) CD127/PD1 subsets of HCV epitope-specific CD8+ T cells (n=7) were analyzed for differential expression of CD122. Statistical significance was assessed by Kruskal-Wallis test. (*, P<0.05.) Median with interquartile range is indicated. MFI: median fluorescence intensity.

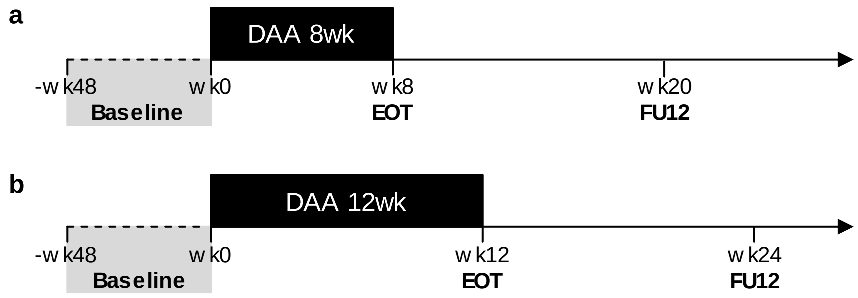

Supplementary Figure 6. DAA treatment scheme and blood sampling during study. Scheme for the two DAA treatment variants are illustrated (8 (**a**) or 12 (**b**) weeks, respectively). Baseline samples were taken before treatment initiation. End of therapy (EOT) was either 8 or 12 weeks after treatment initiation. FU12 is the time point 12 weeks after EOT.

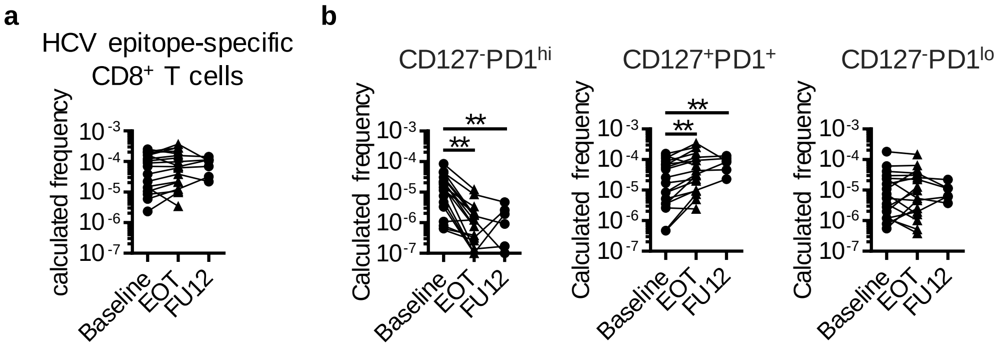

Supplementary Figure 7. Frequencies of HCV-specific CD8+ T cells during therapy. Frequencies of (**a**) HCV epitope-specific CD8+ T cells or (**b**) the indicated CD127/PD1 subset of HCV epitope-specific CD8+ T cells among total CD8+ T cells during DAA therapy was calculated (n=18). Statistical significance was assessed by Friedman test. (**, P<0.01)

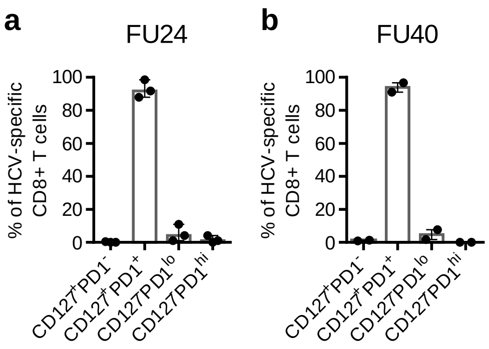

Supplementary Figure 8. CD127/PD1-based heterogeneity of HCV-specific CD8+ T cells at FU24 and FU40. HCV epitope-specific CD8+ T cells were analyzed for phenotype at (a) FU24 (3 HCV-specific CD8+ T-cell responses from 3 patients) and (b) FU40 (2 HCV-specific CD8+ T-cell responses from 1 patient). Median with interquartile range is indicated.

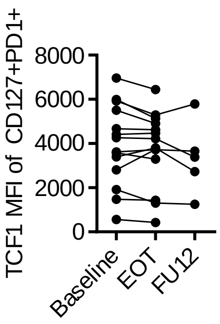

Supplementary Figure 9. TCF1 expression in CD127+PD1+ cells does not change during DAA therapy. The TCF1 MFI (median fluorescence intensity) of CD127+PD1+ HCV epitope-specific CD8+ T cells was analyzed during DAA-mediated HCV elimination (n=14). Statistical significance was assessed by Friedman test.

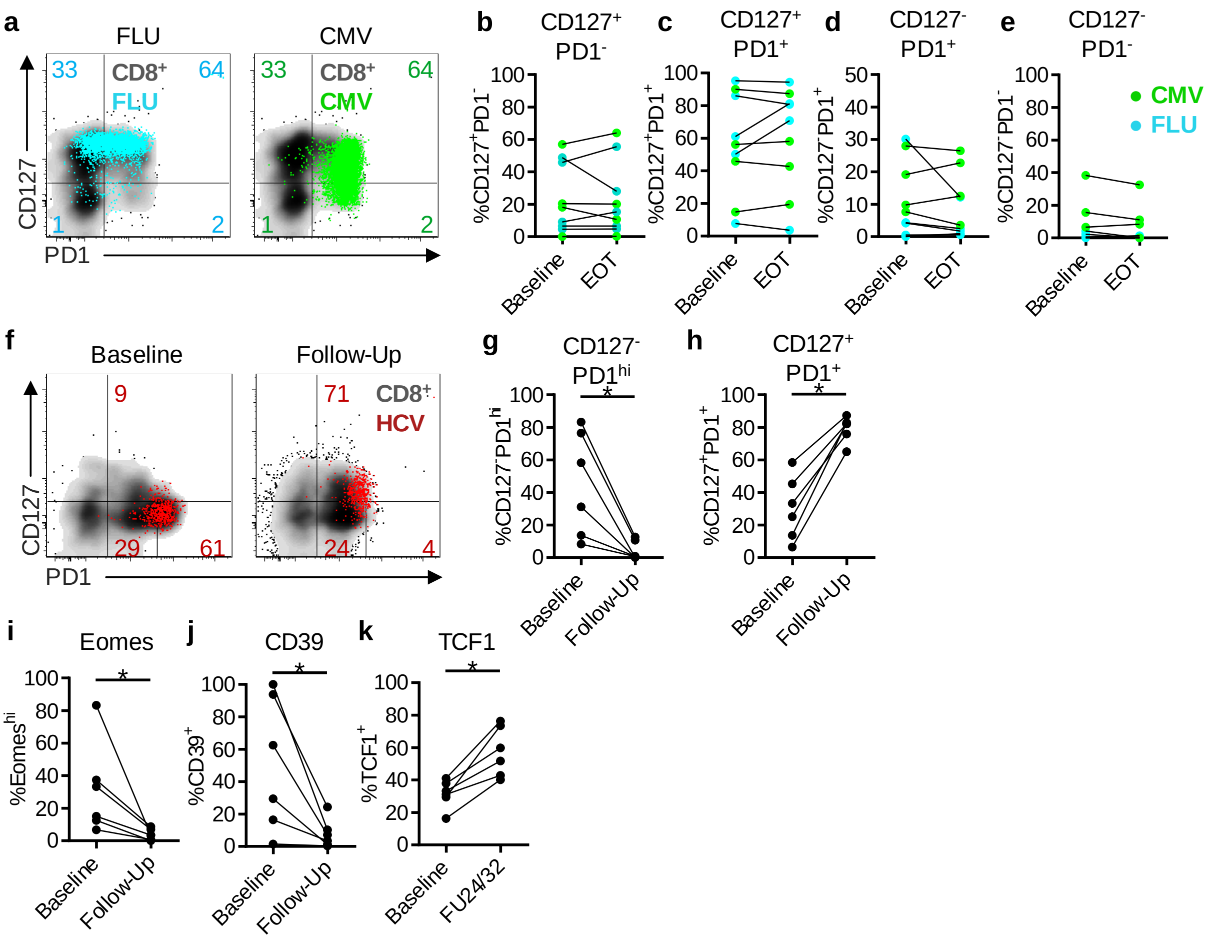

Supplementary Figure 10. Dynamics in T-cell phenotype during and after antigen persistence. (**a-e**) CD127/PD1 co-expression analysis of FLU (n=5) and CMV (n=4) epitope-specific CD8+ T cells in chronically HCV-infected patients (n=6) during DAA-mediated antigen removal. (**a**) Representative dot plots for CD127/PD1 co-expression of FLU (light blue) and CMV (light green) epitope-specific CD8+ T-cell populations. (**b-e**) Analysis of CD127/PD1 subsets of FLU and CMV epitope-specific CD8+ T cells. (**f-k**) Phenotypic analysis of HCV epitope-specific CD8+ T cells during IFNα-based therapy (n=5 with 6 HCV epitope-specific CD8+ T-cell responses). (**f**) Representative dot plots of CD127/PD1 co-expression of HCV epitope-specific CD8+ T cells (red; grey: bulk CD8+ T cells) at baseline and at therapy follow-up. (**g**) CD127-PD1hi and (**h**) CD127+PD1+ subset distribution at baseline and at follow-up. Further depicted is the expression of Eomes (**i**), CD39 (**j**) and TCF1 (**k**). Statistical significance was assessed by Wilcoxon test. (*, P<0.05.)

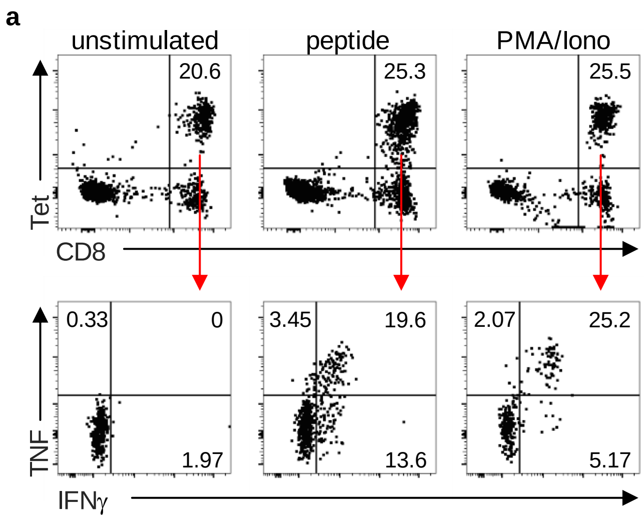

Supplementary Figure 11. Analysis of cytokine production by tetramer-labelled FLU-specific CD8+ T cells. Following HLA-A*02 peptide-specific tetramer enrichment of FLU epitope-specific CD8+ T cells, the enriched sample was either left unstimulated (left panel) or stimulated with FLU epitope-specific peptide (middle panel) or PMA/Ionomycin (right panel) for 5h at 37°. Subsequently, cells were stained for surface molecules and intracellular cytokines and analyzed by flow cytometry. Top panel shows representative dot plots for stability of tetramer staining upon peptide stimulation. Lower panel is gated on tetramer-positive cells and shows expression of IFNγ and TNF upon 5h peptide-specific stimulation.

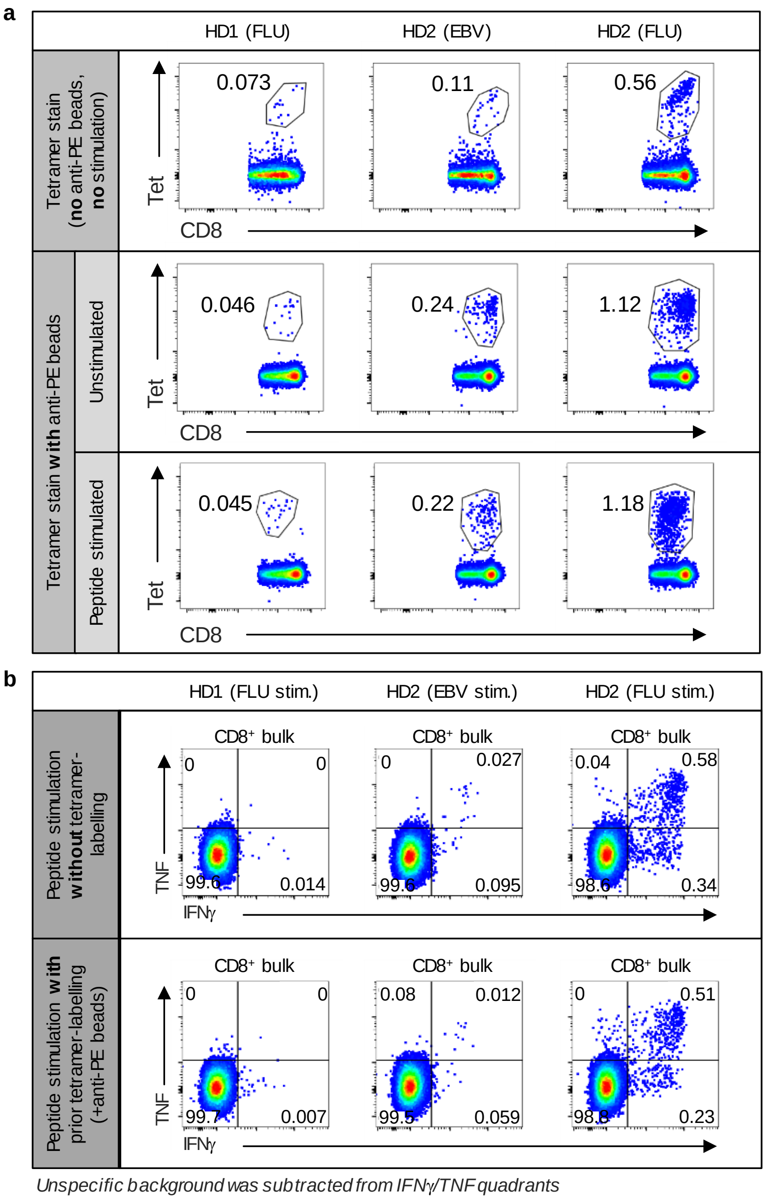

Supplementary Figure 12. Quality of tetramer and cytokine staining after tetramer- and magnetic bead-labeling of virus-specific CD8+ T cells. (**a**) FLU (left and right panel) and EBV (middle panel) epitope-specific CD8+ T cells from healthy donors (HD) (n=2) were labelled with peptide/HLA-A*02 tetramers and subsequently incubated either without (upper panel) or with (middle and lower panel) anti-fluorochrome (anti-PE) microbeads. One part of the peptide/HLA-A*02 tetramer- and microbead-labelled cells was then stimulated with epitope-specific peptides and the peptide/HLA-A*02 tetramer stainings from all three conditions were compared. (**b**) Cytokine production by bulk CD8+ T cells after peptide stimulation was analyzed for cells either without (upper panel) or with (lower panel) prior peptide/HLA-A*02 tetramer/microbead-labelling (epitope-specificity indicated at the top). Frequencies in the quadrants are after background subtraction (unstimulated sample).

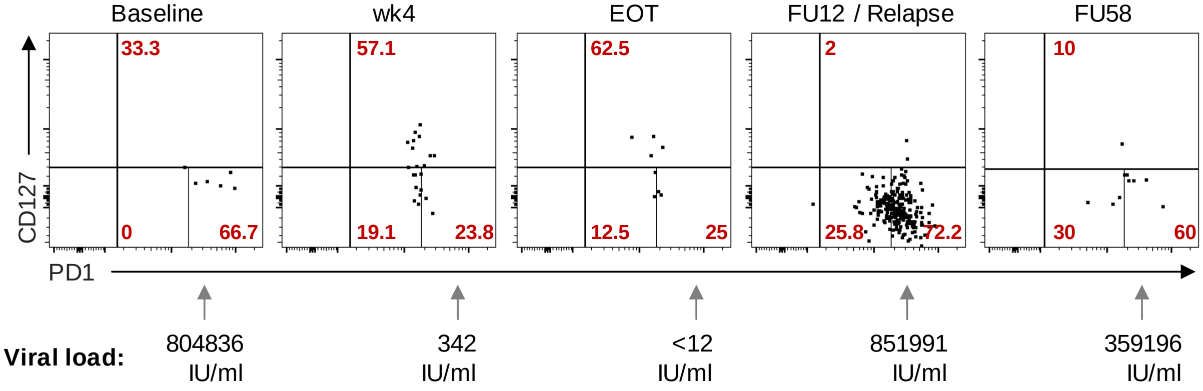

Supplementary Figure 13. CD127/PD1 phenotype of HCV-specific CD8+ T cells in a patient with viral relapse. The HCV viral load at the indicated time points during and after DAA therapy is depicted below.

Supplementary Table 1 Patient characteristics of subjects analyzed for CMV, EBV and FLU epitope-specific CD8+ T cells

Pt	D	Cohort	Treatment	Outcome	gt	analyzed viral epitope	VL	age	sex	CIR
**3**	cHCV	Control	Ledipasvir / Sofosbuvir 12wk	SVR	1a	FLU $M1_{58}$	1.06	52	m	no
**12**	cHCV	Control	Ledipasvir / Sofosbuvir / Ribavirin 12Wk	SVR	1a	CMV $pp65_{495}$	0.04	73	f	no
**41**	cHCV	Control	Ledipasvir / Sofosbuvir 12wk	SVR	1	CMV $pp65_{495}$; FLU $M1_{58}$	5.76	56	m	no
**42**	cHCV	Control	Ledipasvir / Sofosbuvir 12wk	SVR	1b	FLU $M1_{58}$	0.22	66	f	no
**43**	cHCV	Control	Sofosbuvir / Daclatasvir 12wk	SVR	3a	CMV $pp65_{495}$; FLU $M1_{58}$	0.84	42	m	no
**44**	cHCV	Control	Ledipasvir / Sofosbuvir 12wk	SVR	4a	CMV $pp65_{495}$; FLU $M1_{58}$	1.35	53	m	no
**45**	cHCV	Control	-	-	1b	CMV $pp65_{495}$	0.97	65	m	no
**46**	cHCV	Control	-	-	1b	CMV $pp65_{495}$	0.61	56	f	no
**47**	cHCV	Control	-	-	3a	CMV $pp65_{495}$	0.42	63	f	no
**48**	cHCV	Control	-	-	1b	CMV $pp65_{495}$	0.13	59	f	no
**49**	cHCV	Control	-	-	3a	CMV $pp65_{495}$	0.79	43	m	no
**50**	HD	HD	-	-	-	FLU $M1_{58}$; EBV $BMFL1_{280}$	-	28	m	no
**51**	HD	HD	-	-	-	FLU $M1_{58}$	-	27	f	no
**52**	HD	HD	-	-	-	EBV $BMFL1_{280}$	-	25	f	no
**53**	HD	HD	-	-	-	CMV $pp65_{495}$; FLU $M1_{58}$	-	57	m	no
**54**	HD	HD	-	-	-	CMV $pp65_{495}$; FLU $M1_{58;}$ EBV $BMFL1_{280}$	-	63	m	no

Patient characteristics are depicted for the subjects analyzed for CMV, EBV and FLU epitope-specific CD8+ T cells. Pt: Patient; D: Diagnosis; gt: HCV genotype; CIR: cirrhosis; HD: healthy donor; wk: week; SVR: sustained virological response; VL: baseline viral load [IU/ml $x10^6$]; f: female; m: male.

Supplementary Table 2 Patient characteristics of subjects treated with IFNα-based therapy

Pt	D	Cohort	Treatment	Outcome	gt	$NS3_{1073}$	$NS3_{1406}$	VL	age	sex	CIR
**55**	cHCV	IFNα	Peg. IFN-2α / Ribavirin 48wk	SVR	1a	---------	------V---	0.79	72	m	no
**56**	cHCV	IFNα	Peg. IFN-2α / Telaprevir / Ribavirin 48wk	SVR	1a	---------		5.02	44	m	no
**57**	cHCV	IFNα	Peg. IFN-2α / Telaprevir / Ribavirin 48wk	SVR	1a	---------		3.21	53	m	no
**58**	cHCV	IFNα	Peg. IFN-2α / Ribavirin 48wk	SVR	1b	---------		4.60	44	f	no
**59**	cHCV	IFNα	Peg. IFN-2α / Telaprevir / Ribavirin 24wk	SVR	1b	---------		1.54	61	f	no

Patient characteristics of chronically HCV-infected (cHCV) subjects treated with IFNα-based therapy (IFNα cohort). In column $NS3_{1073}$ and $NS3_{1406}$ the viral sequence for each analyzed CD8+ T-cell epitope during chronic HCV infection is shown: (-) indicates a sequence position that corresponds to wild type viral sequence, capital letters show amino acid substitutions varying from wild type sequence. Pt: Patient; D: Diagnosis; gt: HCV genotype; CIR: cirrhosis; Peg.: pegylated; wk: week; SVR: sustained virological response; VL: baseline viral load [IU/ml x10^6] f: female; m: male.

Supplementary Table 3 Primer pairs for viral sequencing

	Epitope	PCR	Forward Primer	Reverse Primer
Genotype 1a	NS31073	1st:	CGTCTGCTCCTGCTTGTGG	ATCCGTGGARTGGCACTCR
		2nd:	ATGTGGCCTCTCCTCCTGC	GCCACCTGGAAGCTCTGGG
	NS31406	1st:	GACAAAAACCARGYGGAGGG	GAGGACCTTCCCCAGYCC
		2nd:	ATAGCAGGGGYAGCCTGC	AGCACAGCCYGCGTCATAGC
	NS52594	1st:	AACCACCTGTGGTCCATGG	TTCATCGGTTGGGGAGGAGG
		2nd:	GGARGAYGTCGTGTGCTGC	TTGCCACATATGGCAGCC
Genotype 1b	NS31073	1st:	GCCGCGATGCCATCATCC	CATTAGAGCGTCTGTTGC
		2nd:	TTGCGGTGGCAGHAGAGC	CGCCCGTGGTGATGGTCC
	NS31406	1st:	ACAAGAACCAGGTCGAGGG	TCTGCTTGAAYTGCTCGG
		2nd:	CCTACYTGAAGGGCTCYTCGGG	GGTGTATTTAGGTAAGCCCG
	NS52594	1st:	TCACAGCTCCCATGYGAGCC	CTTYGCAGCTCGACAGGC
		2nd:	ATGGGCGGRAACATCACCCG	TARAGGGCCATYTTCTCGC

Primer pairs used for sequencing of the indicated epitopes of HCV of genotype 1a and 1b, respectively.
