# Supplementary material for: TCF1+ hepatitis C virus-specific CD8+ T cells are maintained after cessation of chronic antigen stimulation
Source: Nat Commun. 2017 May 3;8:15050. doi: 10.1038/ncomms15050 (PMC5418623; doi:10.1038/ncomms15050)
Supplement: Peer Review File [file ncomms15050-s2.pdf]

Reviewers' comments:

Reviewer #1 (Remarks to the Author):

Wield et al. characterized antigen-specific CD8 T cells in chronic HCV patients, both before and after the antiviral DAA treatment, and identified a TCF1-expressing CD127+PD1+ subset that persisted after the DAA treatment, correlated with improved cytokine production and better expansion. In light of publication of several papers (including two in Nature) on CD8 T cells and chronic infection, this study provides further validation of the concept that there is a subset of less exhausted CD8 T cells with the potential to transition into protective memory CD8 T cells. This is particularly important because these studies were performed in patients with chronic infection. Further the comparison with spontaneous resolvers is informative, and studies of flu-specific CD8 T cells are useful controls. There are several issues that the authors need to improve on.

Major issues:

1. Identification of the TCF1-expressing subset in HCV patients is of interest. Because detection of TCF1 requires intracellular staining, functional characterization of this subset is therefore limited. In light of recent papers showing correlation of TCF1 and CXCR5 expression in the less exhausted, progenitor cells, the authors should determine the correlation between TCF1 and CXCR5 in their cohorts, before and after DAA. Ideally Bcl6 expression can be also examined. It may not be possible to repeat on all patients, 3 replicates should be sufficiently informative.

2. The entire study focused on the frequency of different population (except for the in vitro expansion experiments). This is hard to make firm conclusion without cell number counts. The authors claim that TCF1+ subset specifically persisted, however, it is possible that PD1 high subset was specifically eliminated after DAA treatment. The same argument applies to page 12, lines 6-10, where the conclusion is based on correlative, relative abundancy of the TCF1+ subset. This reviewer is aware that the cells were enriched using tetramer. Quantitative measurement is still possible and will be informative to support the authors' conclusion.

3. Memory CD8 T cells undergo longitudinal maturation over time. The authors found that 12 weeks after DAA treatment, the TCF1+ subset showed limited improvement in cytokine production and proliferation capacity in recall response, compared with SpR. For SpR patients, is there information how long after the initial infection? Can the author perform follow up, longer than 12 weeks after DAA and determine if there is better functional improvement?

Minor issue:

1. Another Nature Immunology paper by Yu D and colleagues should be cited.
2. Figure 1a and 1b, the gating of PD1 high population was not consistent, with that in 1b shifted to right.
3. labels in many figure panels were too small.
4. the in vitro expansion experiments seemed to be done in less than optimal conditions. The best expansion is about 1.5 fold after 14 day culture (Figure 6). Are these specific to exhausted cells? How do memory CD8 T cells from acute infection behave under the same condition? Could culture conditions such as cytokine and peptide concentration can be optimized?
5. more info is needed for the one patient in figure 7. Is the patient treated after relapse. Some data showed 70 weeks for viral load (7a), and called FU58, in legend called FU1yr. should be consistent.
6. several times the authors stated "significant relative increase of TCF1 expression" (e.g., page 8, line 34). This is not accurate, what the authors referred to was actually increased in the percentage of TCF1-expressing cells. if they want to make a point on TCF1 expression level, MFI should calculated and compared.
7. page 7, line 5 "homogeneous CD127+PD1+" is an overstatement and not accurate, should be

something like “strongly skewed to …”

Reviewer #3 (Remarks to the Author):

Review of Wieland et al

In these studies Wieland and colleagues address the durability and differentiation state of HCV-specific CD8 T cells upon cure of chronic HCV infection by direct acting antivirals (DAAs). This is an important question. The data presented build on recent insights into subsets of exhausted CD8 T cells and features that might contribute memory potential or durability / recall capacity to a subset of exhausted T cells. Overall, the data presented are generally high quality and compelling, extend recent studies in mice to an important human chronic viral infection, and will be a valuable addition to the field. Despite enthusiasm for the work, there are a number of areas that could be improved and/or clarified.

The authors present an analysis of phenotypic changes in the HCV-specific CD8 T cell populations following SVR in Figure 3. These data demonstrate a reduction in frequency of some subsets that are suggested to be terminal. One wonders whether it is possible to provide some additional information about numerical changes or other data to begin to address the question of selective survival/death versus conversion of phenotype. Does data exist on cell numbers / ul of blood (admittedly this will still be a frequency of sorts, but might add insight). Whether based on numbers or other approaches some additional insights into this question would be valuable.

The cytokine functional data is interesting, but the assay appears suboptimal (admitted by the authors). It is quite surprising that only ~30% of the flu specific CD8 T cells (the positive control) are functional using their assay. One possibility is that the tetramers are identifying cells that are not truly peptide-specific. This seems unlikely given the quality of the tetramer staining, but one does worry about this issue since such “background” staining could have a substantial impact on the phenotypic data (since non-specific tetramer staining often has a CD127+ phenotype). Do the authors have insights for this poor cytokine production (even after PMA/Ion)? What other controls were performed? Do bulk effector memory or TEMRA cells show better function to PMA/Ion? Does the tetramer enrichment or tetramer staining reduce function?

The TCF-1 correlation for proliferative status (i.e. Figure 6) is interesting, but it is unclear whether the correlations in Figure 6b/c are with the phenotype of the cells at the beginning of the assay or at the end. More importantly, the causal relationship of TCF-1 in this setting remains unclear. Have the authors considered attempting to express TCF-1 in TCF-1 negative cells or knock down TCF-1 in the TCF-1+ subset? Directly testing the prediction of their data would be nice.

Minor concerns:

In Figure 4f have the authors examined TCF-1 expression in the PD-1+CD127+ vs CD127+PD-1- subsets? In addition to percent positive, what does the MFI of TCF-1 look like in these subsets? Also, how does TCF-1 expression compare in subsets defined by Eomes expression?

Figure 7 presents an interesting anecdote. While the difficulties in analyzing such examples is fully acknowledged, the lack of data between weeks 12 and 24 after DAA initiation is unfortunately and would have provided likely informative insights. Moreover, the number of events analyzed at EOT is very low and raises some concerns. It is certainly worth reporting these data, but if even a single additional such example were available confidence would be greatly improved.

In Supplemental Figure 2, how does T-bet MFI compare to naïve CD8 T cells? Is cHCV low, but still

above naïve? If so, how much above?

For Supplemental Figure 3, it might be worth presenting the CD122 data as MFI also given the potential challenge of identifying truly positive and negative cells for CD122.

Response to referees - Manuscript NCOMMS-16-21815-T

"TCF1 expressing Hepatitis C virus-specific CD8+ T cells are maintained after cessation of chronic antigen stimulation"

We thank the reviewers for their valuable comments that helped to significantly improve our manuscript. Following the reviewers suggestions, we have performed additional experiments and revised the manuscript accordingly. In the following, you will find a point-by-point rebuttal to the reviewers' comments.

REVIEWER 1

Reviewer 1 – Major issue 1:

"Identification of the TCF1-expressing subset in HCV patients is of interest. Because detection of TCF1 requires intracellular staining, functional characterization of this subset is therefore limited. In light of recent papers showing correlation of TCF1 and CXCR5 expression in the less exhausted, progenitor cells, the authors should determine the correlation between TCF1 and CXCR5 in their cohorts, before and after DAA. Ideally Bcl6 expression can be also examined. It may not be possible to repeat on all patients, 3 replicates should be sufficiently informative."

Indeed, recent publications in the LCMV mouse model revealed the existence of a CXCR5+ LCMV-specific CD8+ T-cell population in chronic infection that resides mainly in the white pulp of lymphoid organs[1, 2, 3]. These CXCR5+ virus-specific CD8+ T cells are characterized by expression of TCF1, PD1 and BCL6 and function as a "stem cell-like" population for exhausted virus-specific CD8+ T cells during chronic viral infection. We completely agree with the reviewer that the question for co-expression of CXCR5 is highly relevant in our study, since we also report the existence of a TCF1+ PD1+ virus-specific CD8+ T-cell population. To address this point we analyzed PBMCs derived from 5 chronically HCV-infected patients who were treated with DAAs for co-expression of TCF1 and CXCR5 on HCV epitope-specific CD8+ T cells 24 weeks after end of treatment (FU24: follow up 24). The majority of HCV epitope-specific CD8+ T cells expressed TCF1 at FU24, however, we could not detect CXCR5+ HCV epitope-specific CD8+ T cells (3 representative HCV epitope-specific CD8+ T-cell responses are shown) (**Rebuttal Figure 1a**). Of note, we also analyzed CXCR5 expression on total CD8+ T cells derived from PBMCs and did not detect a significant CXCR5+ CD8+ T-cell population (**Rebuttal Figure 1b**). Thus, one explanation for the lack of CXCR5+ HCV epitope-specific CD8+ T cells could be that we analyzed PBMCs and not lymphoid tissue-derived cells.

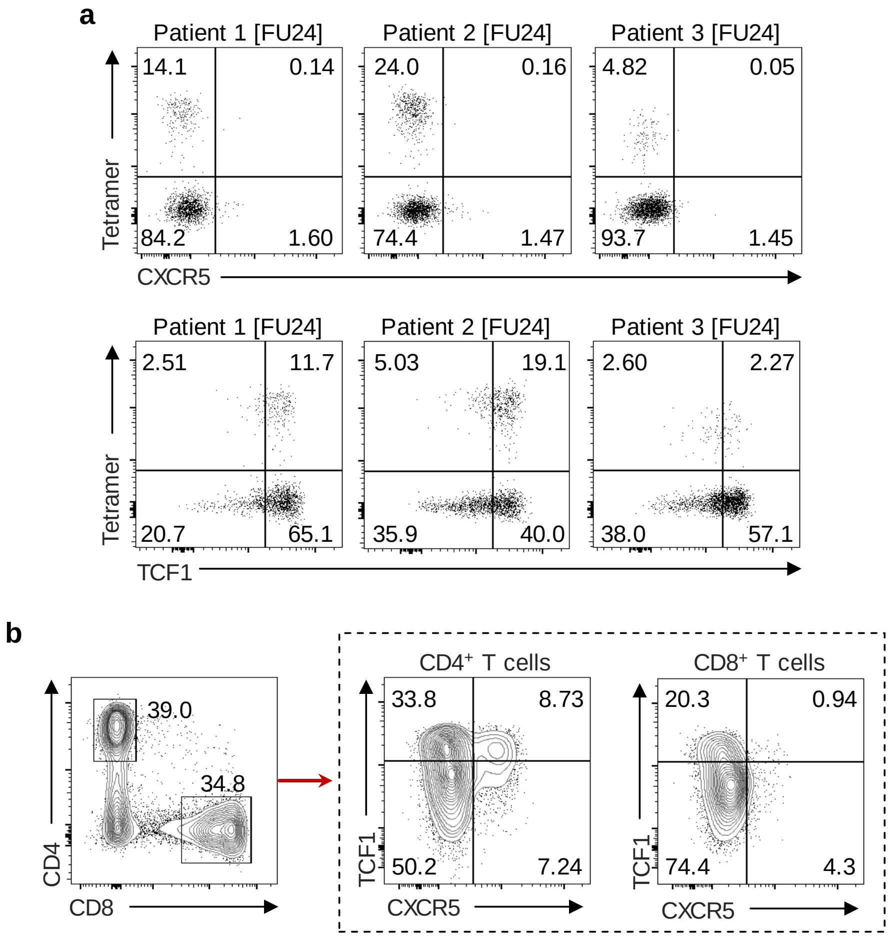

Rebuttal Figure 1 *PBMC-derived TCF1+ HCV epitope-specific CD8+ T cells lack CXCR5 expression at FU24. (**a**) Expression of CXCR5 (upper panel) and TCF1 (lower panel) was analyzed on HCV epitope-specific CD8+ T cells at FU24. Representative data are shown. (**b**) Representative dot plot of PBMCs analyzed for CXCR5 and TCF1 expression on CD4+ and CD8+ T cells.*

Reviewer 1 – Major issue 2

Reviewer 1: "The entire study focused on the frequency of different population (except for the in vitro expansion experiments). This is hard to make firm conclusion without cell number counts. The authors claim that TCF1+ subset specifically persisted, however, it is possible that PD1 high subset was specifically eliminated after DAA treatment. The same argument applies to page 12, lines 6-10, where the conclusion is based on correlative, relative abundancy of the TCF1+ subset. This reviewer is aware that the cells were enriched using tetramer. Quantitative measurement is still possible and will be informative to support the authors' conclusion."

We agree with the reviewer that it is very important to have quantitative information to determine whether a subset of HCV epitope-specific CD8+ T cells is specifically maintained or depleted. Unfortunately, since

lymphocyte counting is not routinely being done in our laboratories before PBMC isolation, we do not have data about lymphocyte counts / ul of patients' blood and thus we cannot provide quantitative measurements. However, we set out to address this important issue by two separate approaches. First, we analyzed the frequency of HCV epitope-specific CD8+ T cells (%Tetramer+ of CD8+ T cells). While the overall frequency of HCV epitope-specific CD8+ T cells did not change significantly during therapy, the frequency of CD127-PD1hi HCV epitope-specific CD8+ T cells strongly decreased (**Rebuttal Figure 2a** and **2b**, also Supplementary Figure 6 in the revised manuscript). In agreement with that, the frequency of CD127+PD1+ HCV epitope-specific CD8+ T cells increased. Second, to determine whether the PD1hi subset is specifically eliminated, we analyzed the abundance of active caspase-8 in the different CD127/PD1 subsets. Importantly, the CD127-PD1hi subset harbored significantly more active caspase-8 than the CD127+PD1+ subset of HCV epitope-specific CD8+ T cells which together with low BCL2 expression indicates higher susceptibility to apoptotic cell death (**Rebuttal Figure 2c** and **d**, also see Figure 2g of the revised manuscript). Together, these data support the hypothesis of a specific loss of CD127-PD1hi HCV epitope-specific CD8+ T cells after cessation of chronic antigen stimulation. The new results are now discussed on page 8 of the revised manuscript.

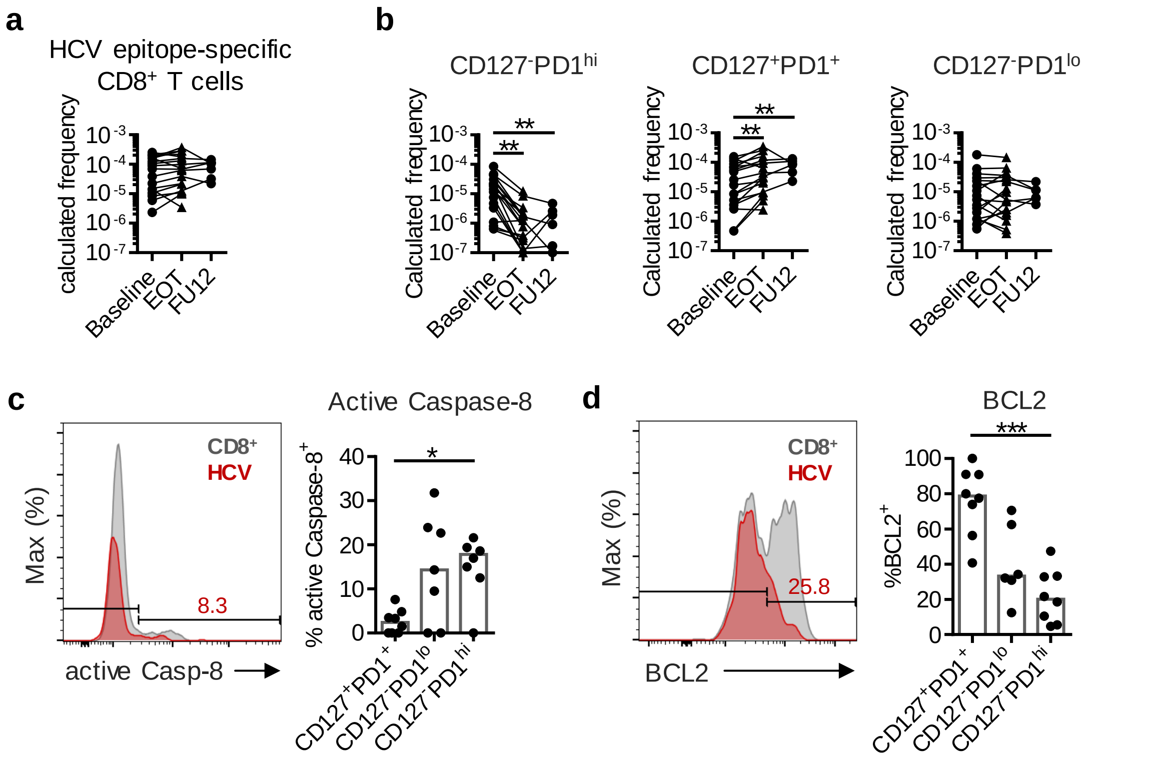

Rebuttal Figure 2 *(Supplementary Figure 6 and Figure 2f,g of the revised manuscript) Loss of CD127-PD1hi HCV epitope-specific CD8+ T cells during therapy. Frequency of (**a**) HCV epitope-specific CD8+ T cells or (**b**) the indicated CD127/PD1 subset of HCV epitope-specific CD8+ T cells among total CD8+ T cells during DAA therapy was calculated (n=18). (**c**) Active caspase-8 and (**d**) expression of BCL2 was determined in the CD127/PD1 subsets of HCV epitope-specific CD8+ T cells. Statistical significance was assessed by Friedman test (**a**,**b**) or Kruskal-Wallis test (**c**,**d**).*

Reviewer 1 – Major issue 3

"Memory CD8 T cells undergo longitudinal maturation over time. The authors found that 12 weeks after DAA treatment, the TCF1+ subset showed limited improvement in cytokine production and proliferation capacity in recall response, compared with SpR. For SpR patients, is there information how long after the initial infection? Can the author perform follow up, longer than 12 weeks after DAA and determine if there is better functional improvement?"

We thank the reviewer for this important question. Of note, we have no information about the time point of initial infection in most cases of our SpR cohort. However, for four of the analyzed patients, early documentations by physicians indicate the year of HCV clearance (**Rebuttal Table 1**, now implemented in Table 1 of the revised manuscript). In most spontaneous resolvers a previous HCV infection was detected as an incidental finding during a visit at the University Hospital Freiburg (anti-HCV IgG positive, HCV-RNA negative).

To determine the question whether T-cell changes proceed in the long-term after DAA-mediated HCV elimination, we analyzed HCV-specific CD8+ T cells from additional patient samples at the latest follow-up visits available. As monitoring of the patients during DAA therapy typically ends at FU24, we only can provide data from 3 samples at FU24 and 2 samples at FU40. The phenotype of HCV epitope-specific CD8+ T cells at FU24 and FU40 seem to remain CD127+PD1+ (**Rebuttal Figure 3a** and **3b**). We also performed expansion experiments of HCV epitope-specific CD8+ T cells from baseline to the latest follow-up visits available to analyze their proliferative capacity beyond end of therapy (illustrated in **Rebuttal Figure 3c**). From these data, we cannot yet conclude whether HCV epitope-specific CD8+ T cells acquire a different phenotype over a very long period of many years (e.g. loss of PD1 expression as seen in spontaneous resolvers). However, a dominant epigenetic imprinting (like described by *Pauken et al., Science 2016*[4]) might prevent full memory formation even long-term after antigen clearance. This important issue needs to be addressed in future studies.

Pt	D	Cohort	Approx. time after primary HCV infection	age	sex	CIR
**26**	SpR	SpR	22 years	43	m	no
**27**	SpR	SpR	unknown	39	f	no
**28**	SpR	SpR	unknown	25	m	no
**29**	SpR	SpR	unknown	67	f	no
**30**	SpR	SpR	unknown	38	f	no
**31**	SpR	SpR	unknown	23	m	no
**32**	SpR	SpR	unknown	54	m	no
**33**	SpR	SpR	unknown	57	f	no
**34**	SpR	SpR	unknown	42	m	no
**35**	SpR	SpR	12 years	39	f	no
**36**	SpR	SpR	19 years	37	m	no
**37**	SpR	SpR	unknown	38	m	no
**38**	SpR	SpR	unknown	60	f	no
**39**	SpR	SpR	unknown	54	f	no
**40**	SpR	SpR	13 years	33	m	no

Rebuttal Table 1 *(implemented in Table 1 of the revised manuscript) Patient characteristics of spontaneous resolvers is shown. If known, the approximate time after primary HCV infection is indicated (red arrow).*

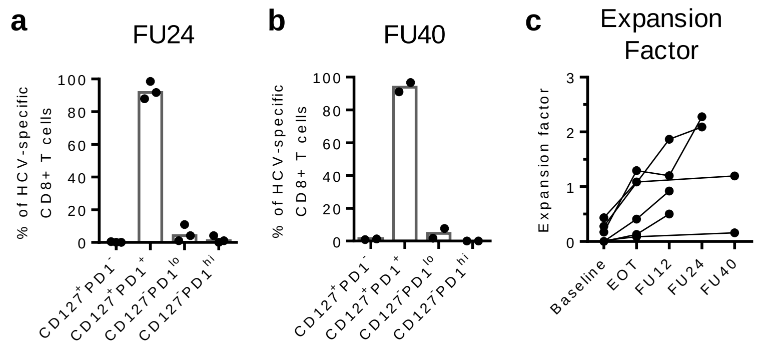

Rebuttal Figure 3 *Phenotypic and functional analysis of HCV epitope-specific CD8+ T cells at FU24 and FU40. HCV epitope-specific CD8+ T cells were analyzed for phenotype at (**a**) FU24 (3 HCV-specific CD8+ T-cell responses from 3 patients) and (**b**) FU40 (2 HCV-specific CD8+ T-cell responses from 1 patient). Moreover, proliferative capacity was determined (**c**).*

Reviewer 1 – Minor issue 1:

Another Nature Immunology paper by Yu D and colleagues should be cited.

We apologize for the missing citation. Yu D. and colleagues are now cited on page 4 of the revised manuscript.

Reviewer 1 – Minor issue 2:

“Figure 1a and 1b, the gating of PD1 high population was not consistent, with that in 1b shifted to right.”

In our flow cytometry experiments fluorescence intensities slightly vary between samples from different patients (stained at the same day) or when samples are stained on different days. With respect to these patient variations and technical fluctuations, we aimed to standardize our gating by setting the PD1hi gates at the upper end of the patients’ bulk CD8+ T-cell population. To avoid confusion on this issue, we exchanged the dot plot in Figure 1a of the revised manuscript (see **Rebuttal Figure 4a**). The dot plots in Figure 1 now both derive from one patient sample that simultaneously harbored two different HCV epitope-specific CD8+ T-cell responses (one that recognizes the autologous viral epitope (**Rebuttal Figure 4a**) and one that does not recognize the viral epitope due to viral escape (**Rebuttal Figure 4b**)).

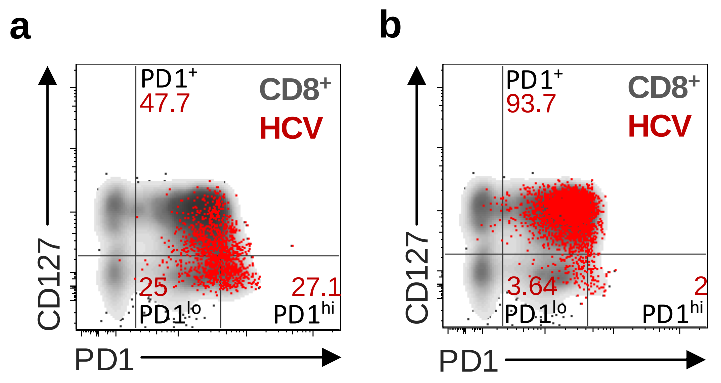

Rebuttal Figure 4 *(now Figure 1 of the revised manuscript) Persistent antigen recognition drives heterogeneity of HCV epitope-specific CD8+ T cells. (**a** and **b**) Subset definition, specifically CD127+PD1+, CD127-PD1lo and CD127-PD1hi cells, is depicted in the representative flow cytometry dot plots. (**a**) HCV-specific CD8+ T-cell response of a chronically HCV-infected patient that recognizes viral antigen. (**b**) HCV-specific CD8+ T-cell response of a chronically HCV-infected patient that does not recognize viral antigen due to viral escape mutations. (red: HCV epitope-specific CD8+ T cells; grey: corresponding bulk CD8+ T cells of the appropriate patient).*

Reviewer 1 – Minor issue 3:

“Labels in many figure panels were too small.“

We increased the font size of our figures to improve readability.

Reviewer 1 – Minor issue 4:

"The in vitro expansion experiments seemed to be done in less than optimal conditions. The best expansion is about 1.5 fold after 14 day culture (Figure 6). Are these specific to exhausted cells? How do memory CD8 T cells from acute infection behave under the same condition? Could culture conditions such as cytokine and peptide concentration can be optimized?"

It is important to note that the term "expansion *factor*" in our study is being used to facilitate logarithmic depiction in the figures:

"Expansion *factor*" = log[expansion *index*+1],

- expansion *index*: fold-increase of virus-specific CD8+ T cells from day 0 to day 14 *in vitro*
- [+1]: allows plotting of zero-values on a logarithmic scale (if cells did not expand at all)

For example, in Figure 6a the highest expansion *index* (fold increase of virus-specific CD8+ T cells) achieved by our *in vitro* conditions was about 1500. However, we agree that the used term "expansion factor" might be misleading and therefore describe the term in more detail in the revised legend of Figure 6 ("The expansion factor is the logarithmic fold-increase in absolute numbers of HCV epitope-specific CD8+ T cells from day 0 to day 14 of *in vitro* culture"). Further details are given in the methods section of the revised manuscript.

Reviewer 1 – Minor issue 5:

"More info is needed for the one patient in figure 7. Is the patient treated after relapse. Some data showed 70 weeks for viral load (7a), and called FU58, in legend called FU1yr. should be consistent."

We thank the reviewer for this important point and apologize for not including more information about patient 13 after viral relapse. We have now extended Table 1 in the revised manuscript by the information "untreated after relapse" (patient 13) (see **Rebuttal Table 2**). Also, the figure legend of Figure 7 was corrected and "FU1yr" replaced by "FU58" (see revised Figure legend 7). In Figure 7a the viral load is depicted as a timeline from baseline to week 70 after treatment initiation. Week 70 thereby is the FU58 (week 12: end of therapy; week 70: follow-up visit 58 weeks after end of therapy).

**13**	cHCV	DAA	Ledipasvir / Sofosbuvir / Ribavirin 12wk: untreated after relapse	RELAPSE	1a	---------	n.d.	62	m	ye

Rebuttal Table 2 *(part of Table 1 of the revised manuscript) Patient characteristic of patient 13 (HCV relapse) was extended by the term "untreated after relapse".*

Reviewer 1 – Minor issue 6:

"Several times the authors stated "significant relative increase of TCF1 expression" (e.g., page 8, line 34). This is not accurate, what the authors referred to was actually increased in the percentage of TCF1-expressing cells. if they want to make a point on TCF1 expression level, MFI should be calculated and compared."

We have corrected our unprecise wording regarding TCF1 expression versus percent of TCF1 expressing cells on page 8: *"In addition, initiation of DAA therapy led to a significant relative increase of ~~TCF1 expression among~~ TCF1 expressing HCV epitope-specific CD8+ T cells (Fig. 3h,i) that can be ascribed to the increased frequency of CD127+PD1+ HCV epitope-specific CD8+ T cells since TCF1 expression remained stable within the CD127+PD1+ subset (Fig. 3h,j and Supplementary Fig. 7)"* In addition, we extended our data by Supplementary Figure 7 in the revised manuscript showing the MFI of TCF1 on CD127+PD1+ HCV epitope-specific CD8+ T cells during therapy (see **Rebuttal Figure 5**).

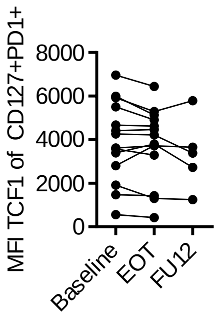

Rebuttal Figure 5 *(Supplementary Figure 7 of the revised manuscript) TCF1 expression by CD127+PD1+ HCV epitope-specific CD8+ T cells does not change during DAA therapy. The TCF1 MFI (median fluorescence intensity) of CD127+PD1+ HCV epitope-specific CD8+ T cells was analyzed during DAA-mediated HCV elimination (n=14). Statistical significance was assessed by Friedman test.*

Reviewer 1 – Minor issue 7:

"page 7, line 5 "homogeneous CD127+PD1+" is an overstatement and not accurate, should be something like "strongly skewed to …""

We also corrected our wording here by replacing the phrase *"do not show a heterogeneous phenotype but rather consist of a homogeneous CD127+PD1+ population"* by *"do not show strong heterogeneity but rather consist of a predominantly CD127+PD1+ population"*).

REVIEWER 3

Reviewer 3 – Major 1:

Reviewer 3: "The authors present an analysis of phenotypic changes in the HCV-specific CD8 T cell populations following SVR in Figure 3. These data demonstrate a reduction in frequency of some subsets that are suggested to be terminal. One wonders whether it is possible to provide some additional information about numerical changes or other data to begin to address the question of selective survival/death versus conversion of phenotype. Does data exist on cell numbers / ul of blood (admittedly this will still be a frequency of sorts, but might add insight). Whether based on numbers or other approaches some additional insights into this question would be valuable."

We agree with the reviewer that it is of major importance to differentiate between cell survival and death versus phenotype conversion. Unfortunately, we cannot give information about cell numbers per ul of blood, since lymphocyte counting is not routinely being done with our patient blood samples before PBMC isolation. However, we addressed this issue by analyzing apoptotic activity in the different CD127/PD1 subsets of HCV epitope-specific CD8+ T cells by determining caspase-8 activity. Importantly, compared to the CD127+PD1+ population the CD127-PD1hi population exhibited significantly more active caspase-8 (**Rebuttal Figure 6a**, now implemented in Figure 2g of the revised manuscript) and reduced BCL2 expression (**Rebuttal Figure 6b**, Figure 2f of the revised manuscript) rendering those cells more prone to apoptosis. These data support the hypothesis of a specific loss of CD127-PD1hi HCV epitope-specific CD8+ T cells after cessation of chronic antigen stimulation which is now discussed on page 8 of the revised manuscript.

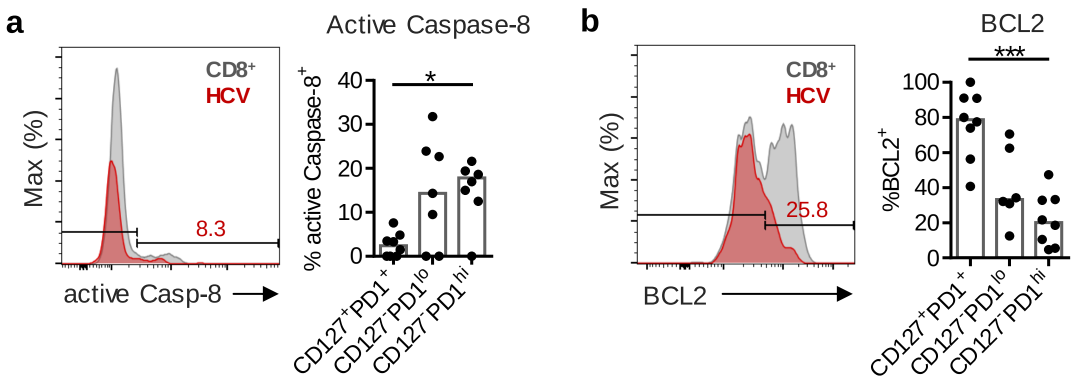

***Rebuttal Figure 6** (now implemented in Figure 2 of the revised manuscript; see Fig. 2g) CD127-PD1hi HCV epitope-specific CD8+ T cells are characterized by higher caspase-8 activity. HCV epitope-specific CD8+ T cells were analyzed for the relative expression of BCL2 (**a**) and active caspase-8 (**b**) with respect to the CD127/PD1 subsets (n=7-8). Statistical significance was assessed by Kruskal-Wallis with Dunn's multiple comparison test.*

Reviewer 3 – Major 2:

"The cytokine functional data is interesting, but the assay appears suboptimal (admitted by the authors). It is quite surprising that only ~30% of the flu specific CD8 T cells (the positive control) are functional using their assay. One possibility is that the tetramers are identifying cells that are not truly peptide-specific. This seems unlikely given the quality of the tetramer staining, but one does worry about this issue since such "background" staining could have a substantial impact on the phenotypic data (since non-specific tetramer staining often has a CD127+ phenotype). Do the authors have insights for this poor cytokine production (even after PMA/Ion)? What other controls were performed? Do bulk effector memory or TEMRA cells show better function to PMA/Ion? Does the tetramer enrichment or tetramer staining reduce function?"

We fully understand the concern of the reviewer and have addressed all suggestions to demonstrate reliability of our data.

During the peptide/MHC class I tetramer enrichment protocol, tetramer-labelled cells are co-incubated with magnetic beads directed against the tetramer-bound fluorochrome (anti-PE or anti-APC). The co-incubation of tetramer-labelled cells with anti-tetramer antibodies already has been described to increase the sensitivity of peptide/MHC class I tetramer stainings[5] by stabilization of the tetramer-MHC link. As shown in **Rebuttal Figure 7a**, we also observed an increased sensitivity to detect virus-specific CD8+ T cells by co-incubation with anti-fluorochrome beads. To demonstrate that the higher frequency of tetramer+ cells was not due to unspecific peptide/MHC class I tetramer binding (especially since non-specific tetramer staining often has a CD127+ phenotype), we analyzed the herein highly relevant CD127/PD1 phenotype on tetramer+ CD8+ T cells detected either with or without anti-fluorochrome bead enrichment. Importantly, the phenotype of detected tetramer+ CD8+ T cells did not differ between the two detection methods. Moreover, we did not observe any accumulation of a typically unspecific CD127/PD1 subset (which could probably be expected to be CD127+PD1- or CD127-PD1-) after peptide/MHC class I tetramer stabilization with anti-PE beads (**Rebuttal Figure 7b-e**). The results indicate that peptide/MHC class I tetramer-binding of CD8+ T cells not specific for the indicated viral epitope is rather unlikely in our protocol. These data are now implemented in Supplementary Figure 1 of the revised manuscript.

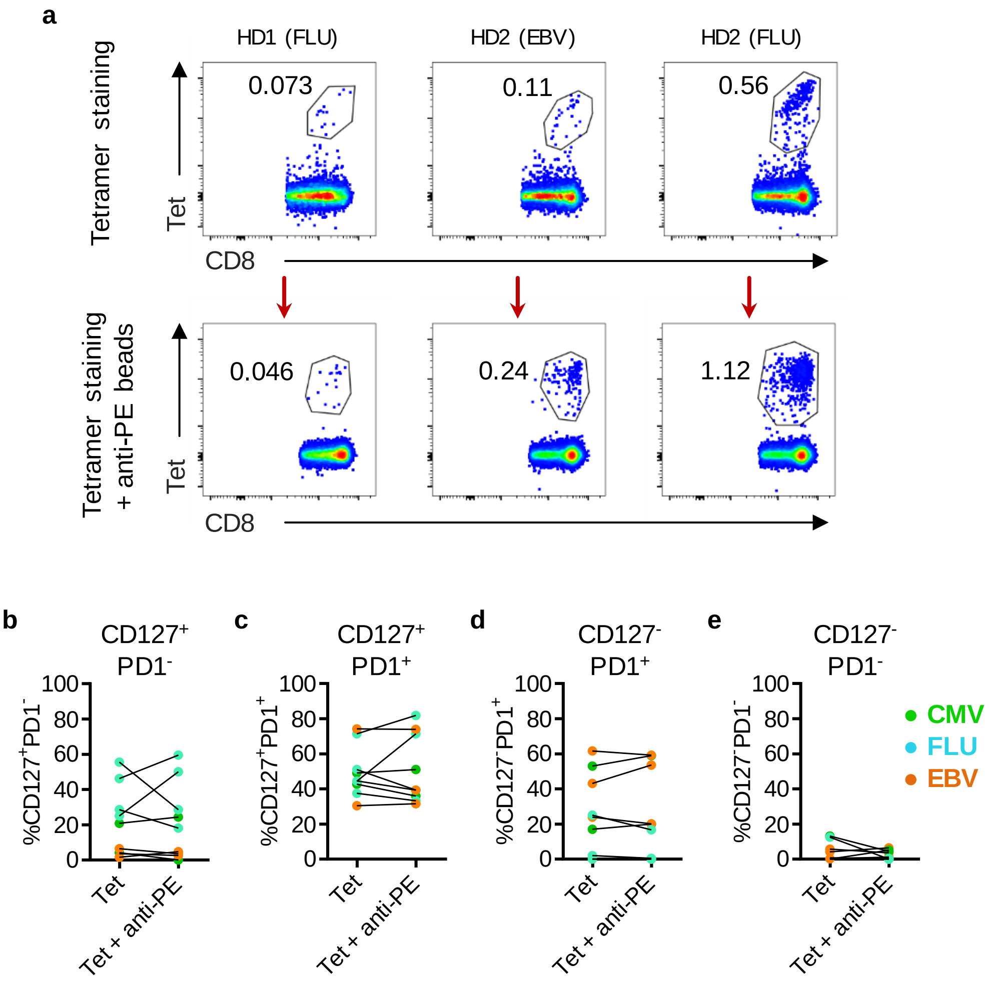

Rebuttal Figure 7 *(Supplementary Figure 1 of the revised manuscript) Peptide/HLA-A*02 tetramer-based enrichment procedure specifically increases detection of virus-specific CD8+ T cells. Peptide/MHC class I tetramer staining was performed with or without subsequent incubation of tetramer-labelled CD8+ T cells with anti-fluorochrome (anti-PE) microbeads. (**a**) Representative dot plots are depicted of three virus-specific CD8+ T-cell response (reaching from low to high frequency) either without anti-PE labelling (upper panel) or with labelling with anti-PE microbeads (lower panel) (2 healthy donors (HD) with 3 virus-specific CD8+ T-cell responses: 2 FLU, 1 EBV). (**b-e**) To exclude unspecific peptide/HLA-A*02 tetramer-labelling and thus enrichment of specific CD127/PD1 subsets, the phenotype of tetramer-labelled virus-specific CD8+ T cells was analyzed before and after co-incubation with anti-fluorochrome beads (9 epitope-specific CD8+ T-cell responses from 5 healthy donors: 4 FLU, 3 EBV, 2 CMV). Statistical significance was assessed by Wilcoxon test.*

Next, we asked whether tetramer-binding reduces peptide-specific cytokine production by CD8+ T cells after peptide-stimulation. Specifically, we stimulated PBMCs harboring virus-specific CD8+ T cells ranging from a very low frequency to a very high frequency (**Rebuttal Figure 8a**) with viral peptide either with or without prior tetramer staining and anti-PE labelling (Tet + anti-PE beads). After 5 hours of peptide-stimulation at 37°C, we analyzed bulk CD8+ T cells for cytokine production. The results show that prior tetramer-labelling only slightly reduces the amount of TNF/IFNγ production by CD8+ T cells (**Rebuttal Figure 8b**). Importantly,

comparable cytokine production could be detected even for virus-specific CD8+ T-cell responses present at low frequencies. Thus, we conclude that cytokine production after peptide stimulation is not severely influenced by prior tetramer staining. These data are now presented by Supplementary Figure 10 of the revised manuscript.

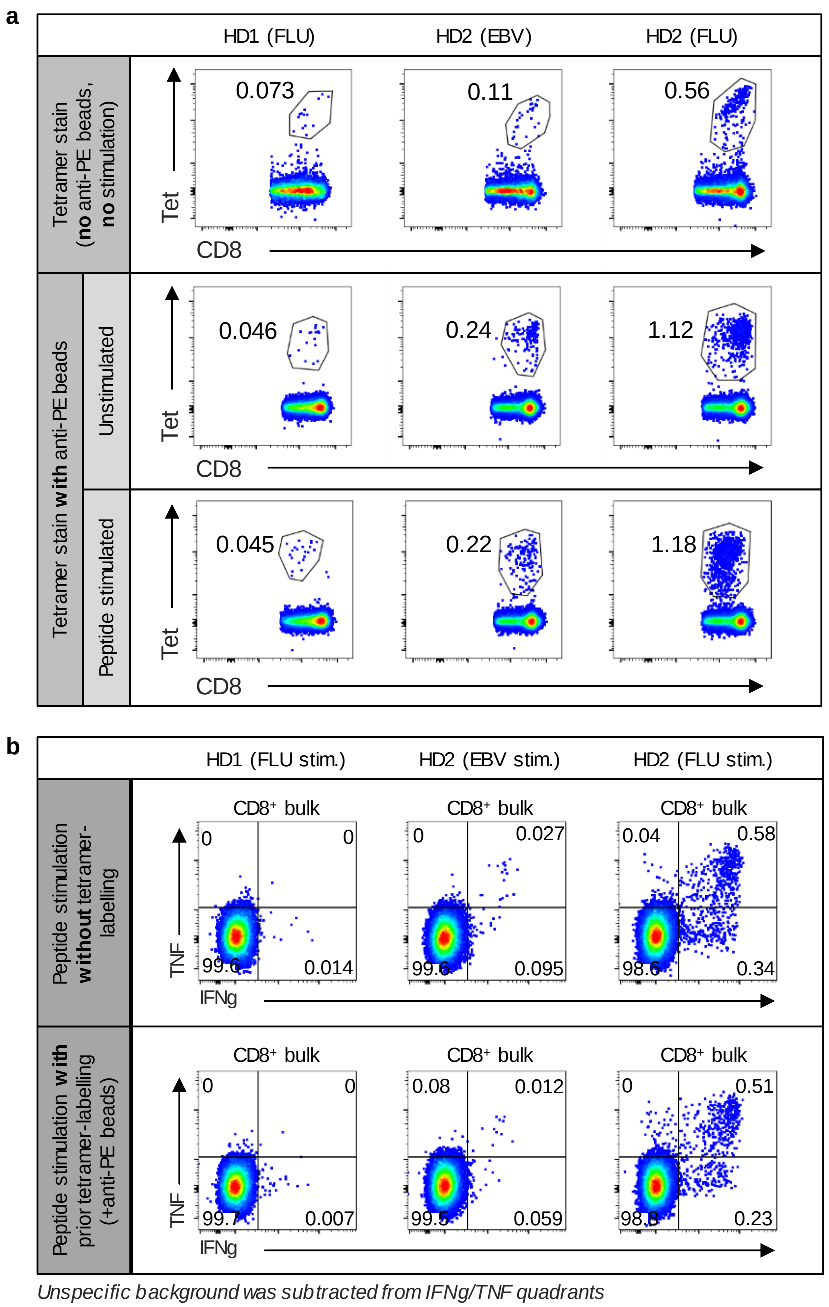

Rebuttal Figure 8 *(now Supplementary Figure 10 of the revised manuscript) Combining peptide/HLA-A*02 tetramer enrichment with epitope-specific peptide stimulation. (**a**) FLU (left and right panel) and EBV (middle panel) epitope-specific CD8+ T cells from healthy donors (HD) (n=2) were labelled with peptide/HLA-A*02 tetramers and subsequently incubated either without (upper panel) or with (middle and lower panel) anti-fluorochrome (anti-PE) microbeads. One part of the tetramer- and microbead-labelled cells was then stimulated with epitope-specific peptides and the tetramer stainings from all three conditions were compared. (**b**) Cytokine production by bulk CD8+ T cells after peptide stimulation was analyzed for cells either without (upper panel) or with (lower panel) prior tetramer/microbead-labelling (epitope-specificity indicated at the top). Frequencies in the quadrants are after background subtraction (unstimulated sample).*

To address the reviewers concern about the poor cytokine production by FLU epitope-specific CD8+ T cells in our cytokine assay we determined the maximum potential of CD8+ T cells to produce cytokines (without prior tetramer staining). After 5 hours of PMA/Ionomycin stimulation, only about 30% of CD8+ T cells were positive for IFNγ production (Mdn: 28.3%; IQR: 17.0-40.2%) (**Rebuttal Figure 9**). Of note, most cytokines were produced by the effector memory T-cell fraction (Mdn: 60.6%; IQR: 40.7-71.6%). The central memory T-cell population (that harbors most of FLU epitope-specific CD8+ T cells[6]) showed a median percentage of IFNγ+ cells of 37.4% (IQR: 18.9-45.3%). Noteworthy, other publications using a similar approach to detect cytokine production by FLU epitope-specific CD8+ T cells reported similar results[7]. And finally, a further factor influencing functionality might be the usage of frozen PBMCs.

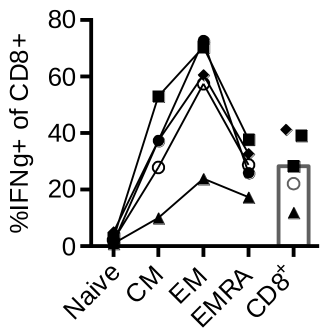

Rebuttal Figure 9 *Cytokine production by CD8+ T cells after 5h PMA/Ionomycin stimulation. PBMCs from 5 healthy donors were stimulated with PMA/Ionomycin for 5h at 37°C and cytokine production in the different CD8+ T-cell subsets (defined by CD45RA and CCR7) was determined.*

In sum, we are convinced that our results are not influenced by unspecific peptide/MHC class I tetramer binding and that our assay is sensitive enough to detect cytokine production by non-exhausted T cells even for T-cell responses present at very low frequencies.

Reviewer 3 – Major 3:

"The TCF-1 correlation for proliferative status (i.e. Figure 6) is interesting, but it is unclear whether the correlations in Figure 6b/c are with the phenotype of the cells at the beginning of the assay or at the end. More importantly, the causal relationship of TCF-1 in this setting remains unclear. Have the authors considered attempting to express TCF-1 in TCF-1 negative cells or knock down TCF-1 in the TCF-1+ subset? Directly testing the prediction of their data would be nice."

We apologize for the unprecise wording and specified our description about the correlation of expansion factor and T-cell phenotype in the revised Figure legend 6: *"Correlation analyses of the expansion factor*

with the abundance of memory-like CD127+PD1+ (b) or TCF1+ (c) HCV epitope-specific CD8+ T cells *<u>at day 0 of the assay</u>".* Moreover, we totally support the idea of Reviewer 3 for TCF1 knock down or overexpression experiments to demonstrate the causal relationship between TCF1 and proliferative capacity. However, this is a major challenge to address in primary human cells, especially due to restricted samples with limited cell numbers. Unfortunately, T-cell clones of the corresponding patients are not available. Due to the difficulty of demonstrating this causality in the human system, we chose a careful wording and only state a correlation (instead of causality) between TCF1 and proliferative capacity. Noteworthy, a role of TCF1 in T-cell proliferative capacity has previously been reported in the mouse model[8].

Reviewer 3 – Minor 1:

"In Figure 4f have the authors examined TCF-1 expression in the PD-1+CD127+ vs CD127+PD-1- subsets? In addition to percent positive, what does the MFI of TCF-1 look like in these subsets? Also, how does TCF-1 expression compare in subsets defined by Eomes expression?"

TCF1 expression did not significantly differ between the main sub-populations of HCV epitope-specific CD8+ T cells that can be found after spontaneous HCV resolution (CD127+PD1+ versus CD127+PD1-) (**Rebuttal Figure 10a**). Regarding TCF1 expression in HCV epitope-specific CD8+ T cells differentiated by Eomes expression, our data show that $Eomes^{hi}$ cells express significantly less TCF1 compared to the $Eomes^{dim}$ population (**Rebuttal Figure 10b**).

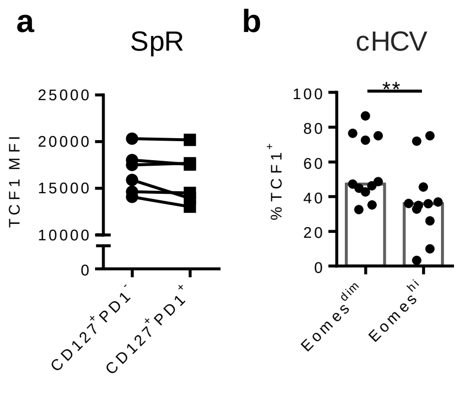

***Rebuttal Figure 10** (**a**) TCF1 expression was compared between CD127+PD1- and CD127+PD1+ HCV-specific CD8+ T cells of spontaneous HCV resolvers (6 HCV-specific CD8+ T-cell responses from 5 SpR patients). (**b**) TCF1 expression was determined on Eomeshi and Eomesdim HCV-specific CD8+ T cells during chronic HCV infection (11 HCV-specific CD8+ T-cell responses from 8 patients). Statistical significance in (**a**) and (**b**) was assessed by Wilcoxon test. (**, P<0.01)*

Reviewer 3 – Minor 2:

"Figure 7 presents an interesting anecdote. While the difficulties in analyzing such examples is fully acknowledged, the lack of data between weeks 12 and 24 after DAA initiation is unfortunately and would have provided likely informative insights. Moreover, the number of events analyzed at EOT is very low and raises some concerns. It is certainly worth reporting these data, but if even a single additional such example were available confidence would be greatly improved."

We agree with the reviewers concerns that we only can show one relapse patient with a low number of detectable HCV epitope-specific CD8+ T cells at the end of therapy. Due to the very high efficiency of DAA therapy, patients with HCV relapse are very rare. Our overall cohort of HCV-infected patients receiving DAAs who were monitored during this study includes about 370 patients. However, only one of these patients who fitted the criteria to be included in this study showed an HCV relapse. Thus, we hope to increase the readers' confidence by presenting all phenotypical analysis we could perform on the available patient samples. These data show that the phenotypical changes during therapy and after viral relapse are closely linked to viral eradication (**Rebuttal Figure 11**; now Supplementary Figure 11 and page 12 of the revised manuscript).

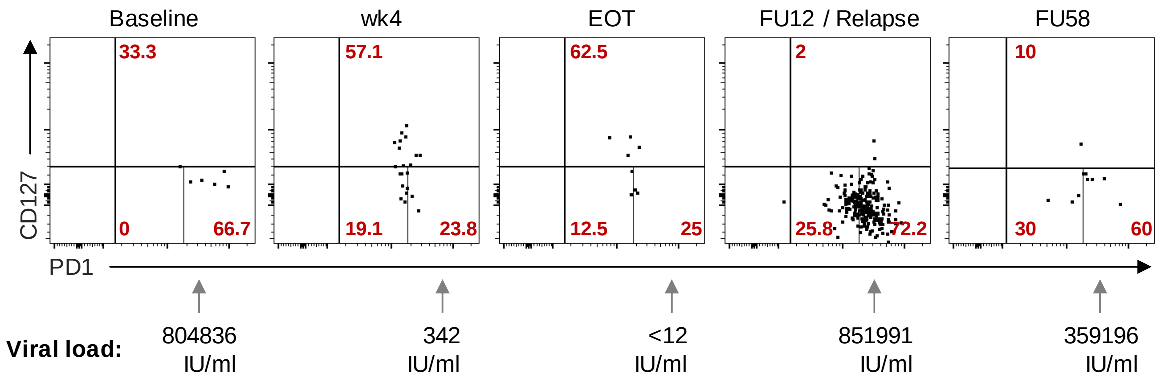

Rebuttal Figure 11 *(now Supplementary Figure 11 of the revised manuscript) CD127/PD1 phenotype of HCV epitope-specific CD8+ T cells in a patient with viral relapse during and after DAA therapy. The HCV viral load at the indicated time points during and after DAA therapy is depicted below.*

Reviewer 3 – Minor 3:

In Supplemental Figure 2, how does T-bet MFI compare to naïve CD8 T cells? Is cHCV low, but still above naïve? If so, how much above?

In addition to comparing T-bet MFI on HCV and CMV epitope-specific CD8+ T cells, we analyzed T-bet on naïve CD8+ T cells of the corresponding patients. As expected by Reviewer 3, naïve CD8+ T cells showed lower T-bet expression compared to HCV epitope-specific CD8+ T cells (T-bet MFI on HCV: Mdn: 2186, IQR: 1584-3572; T-bet MFI on naïve CD8+: Mdn: 822, IQR: 695-1078; T-bet MFI on CMV: Mdn: 7189, IQR: 5634-7854) (**Rebuttal Figure 12**; now Supplementary Figure 3 of the revised manuscript).

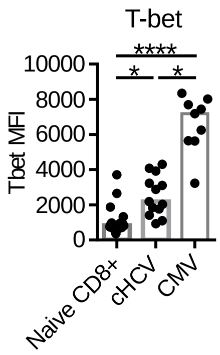

Rebuttal Figure 12 *(Supplementary Figure 3 of the revised manuscript) HCV epitope-specific CD8+ T cells in general exhibit low expression of T-bet. T-bet expression was defined in HCV epitope-specific CD8+ T cells (n=13) and compared to expression in CMV epitope-specific CD8+ T cells (n=9) and naïve CD8+ T cells (n=18) of chronically HCV-infected patients. Statistical significance was assessed by Mann-Whitney test (*, P<0.05.). Bar charts show the median. MFI: median fluorescence intensity.*

Reviewer 3 – Minor 4:

For Supplemental Figure 3, it might be worth presenting the CD122 data as MFI also given the potential challenge of identifying truly positive and negative cells for CD122.

Based on recent findings that LCMV epitope-specific CD8+ T cells do not express CD122 at late time points during chronic viral infection[9], CD122 expression on HCV epitope-specific CD8+ T cells was an unexpected finding. To improve transparency of our data, we extended Supplementary Figure 4 with an example for CD122 gating (**Rebuttal Figure 13a**) and CD122 analysis by MFI (**Rebuttal Figure 13c**). These data are now shown in Supplementary Figure 4 of the revised manuscript.

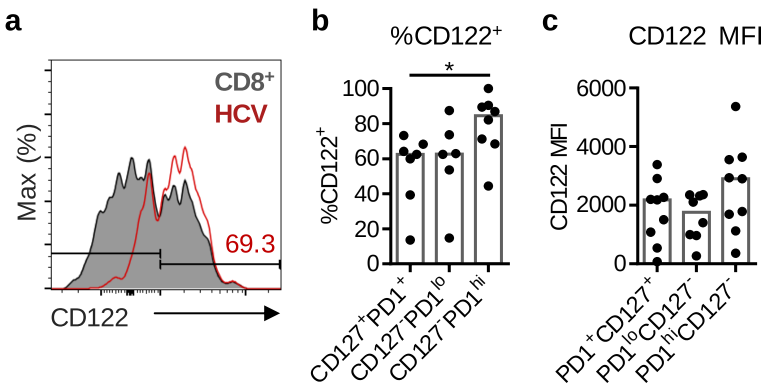

Rebuttal Figure 13 *(now Supplementary Figure 4 of the revised manuscript) The CD127-PD1hi subset of HCV epitope-specific CD8+ T cells shows high CD122 expression. (**a**) Representative flow cytometric histogram plot including gating of CD122 is depicted (red: HCV epitope-specific CD8+ T cells; grey: corresponding bulk CD8+ T cells). (**b**,**c**) CD127/PD1 subsets of HCV epitope-specific CD8+ T cells (n=7) were analyzed for differential expression of CD122. Statistical significance was assessed by Kruskal-Wallis test. (*, P<0.05.) Bar charts show the median. MFI: median fluorescence intensity.*

References:

1. Leong, Y.A. *et al.* CXCR5(+) follicular cytotoxic T cells control viral infection in B cell follicles. *Nat. Immunol.* **17**, 1187-+ (2016).

2. He, R. *et al.* Follicular CXCR5-expressing CD8+ T cells curtail chronic viral infection. *Nature* (2016).

3. Im, S.J. *et al.* Defining CD8+ T cells that provide the proliferative burst after PD-1 therapy. *Nature* (2016).

4. Pauken, K.E. *et al.* Epigenetic stability of exhausted T cells limits durability of reinvigoration by PD-1 blockade. *Science*; 2016.

5. Tungatt, K. *et al.* Antibody Stabilization of Peptide-MHC Multimers Reveals Functional T Cells Bearing Extremely Low-Affinity TCRs. *Journal of Immunology* **194**, 463-474 (2015).

6. Appay, V., van Lier, R.A.W., Sallusto, F. & Roederer, M. Phenotype and Function of Human T Lymphocyte Subsets: Consensus and Issues. *Cytom Part A* **73a**, 975-983 (2008).

7. Pittet, M.J. *et al.* Ex vivo IFN-gamma secretion by circulating CD8 T lymphocytes: Implications of a novel approach for T cell monitoring in infectious and malignant diseases. *Journal of Immunology* **166**, 7634-7640 (2001).

8. Utzschneider, D.T. *et al.* T Cell Factor 1-Expressing Memory-like CD8+ T Cells Sustain the Immune Response to Chronic Viral Infections. *Immunity* **45**, 415-427 (2016).

9. Wherry, E.J. *et al.* Molecular signature of CD8+ T cell exhaustion during chronic viral infection. *Immunity* **27**, 670-684 (2007).

REVIEWERS' COMMENTS:

Reviewer #1 (Remarks to the Author):

The authors have carefully addressed the issues raised by this reviewer and the third reviewer. given the limitation of human studies, there are unsolved questions, which do not necessarily negate the authors' conclusions.

In rebuttal figure 1, they showed that the TCF1+ cells were not associated with increased CXCR5 expression. Although it is negative data, I would think this is useful information and recommend inclusion as a supplemental figure and describe in the text.

Overall, this is a very performed study that fills an important knowledge gap in chronic infection of humans. I support publication of this manuscript.

Reviewer #3 (Remarks to the Author):

The authors have address all of my concerns. This is a comprehensive and important paper for the field. My only minor suggestion is for the authors to perhaps considering including some of the rebuttal figures in the supplement of this manuscript. While some data are not contained in the supplement, some other very nice data was presented only for the reviewers and might be valuable for the field. I defer to the authors here. Just a suggestion.
